# Genome-wide association study reveals sex-specific genetic architecture of facial attractiveness

**DOI:** 10.1371/journal.pgen.1007973

**Published:** 2019-04-04

**Authors:** Bowen Hu, Ning Shen, James J. Li, Hyunseung Kang, Jinkuk Hong, Jason Fletcher, Jan Greenberg, Marsha R. Mailick, Qiongshi Lu

**Affiliations:** 1 Department of Statistics, University of Wisconsin-Madison, Madison, WI, United States of America; 2 Department of Psychology, University of Wisconsin-Madison, Madison, WI; 3 Waisman Center, University of Wisconsin-Madison, Madison, WI, United States of America; 4 La Follette School of Public Affairs, University of Wisconsin–Madison, Madison, WI; 5 Department of Sociology, University of Wisconsin–Madison, Madison, WI; 6 School of Social Work, University of Wisconsin–Madison, Madison, WI; 7 Department of Biostatistics and Medical Informatics, University of Wisconsin-Madison, Madison, WI, United States of America; University of Pittsburgh School of Dental Medicine, UNITED STATES

## Abstract

Facial attractiveness is a complex human trait of great interest in both academia and industry. Literature on sociological and phenotypic factors associated with facial attractiveness is rich, but its genetic basis is poorly understood. In this paper, we conducted a genome-wide association study to discover genetic variants associated with facial attractiveness using 4,383 samples in the Wisconsin Longitudinal Study. We identified two genome-wide significant loci, highlighted a handful of candidate genes, and demonstrated enrichment for heritability in human tissues involved in reproduction and hormone synthesis. Additionally, facial attractiveness showed strong and negative genetic correlations with BMI in females and with blood lipids in males. Our analysis also suggested sex-specific selection pressure on variants associated with lower male attractiveness. These results revealed sex-specific genetic architecture of facial attractiveness and provided fundamental new insights into its genetic basis.

## Author summary

Facial attractiveness is a complex human trait well integrated into people’s daily life experience with profound influence on human behavior. Despite being widely studied in sociology, psychology, and related fields, its genetic basis remains poorly understood. Using carefully-measured facial attractiveness and dense genotyping data from the Wisconsin Longitudinal Study, we identified novel genes for facial attractiveness, assessed the selection signature, and dissected the shared genetic architecture between facial attractiveness and various human traits. Interestingly, sex-specific genetic architecture of facial attractiveness was a recurrent pattern observed in almost all our analyses. Our results provided new insights into the genetic basis of facial attractiveness and have broad implications for the complex relationships between attractiveness and various human traits.

## Introduction

Facial attractiveness is a complex human trait of great interest in sociology, psychology, and related fields due to its profound influence on human behavior. Although variability exists across individuals and cultures, it has been suggested that some commonly agreed cues are used by people everywhere to judge facial beauty [1, 2]. As a trait that is well integrated into people’s daily life experience, it is unsurprising that facial attractiveness influences a variety of sociological outcomes. Studies have suggested that facial attractiveness is associated with job-related outcomes [3–6], academic performance [7], and economic mobility [8]. It affects human psychological adaptations and serves as an important influence of mate preference and reproductive success [9–14]. Even attractive babies receive more nurturing from their mothers than unattractive babies [15]. Further, people all over the world highly prize beauty. The annual revenue of the cosmetic industry is around 18 billion dollars in the US [16]. Fashion and beauty dominate daily discussions on traditional media as well as social media posts. Understanding the perception of attractiveness is a great interest in both academia and industry.

The genetic basis of facial attractiveness may provide new and mechanistic insights into this complex human trait. Evidence suggested that attractiveness is heritable and genetic variations explain a substantial fraction of its variability [17, 18]. However, no genetic variant or gene underlying the biology of facial attractiveness has been identified to date. Our understanding of its genetic architecture is certainly far from complete. In this study, we utilized data from the Wisconsin Longitudinal Study (WLS), a longitudinal study of a 1/3 random sample of over ten thousand Wisconsin high school graduates in 1957. Facial attractiveness in WLS was measured by 12 coders using an 11-point rating scale based on each individual’s 1957 high school yearbook photo. These well-characterized facial attractiveness data from WLS have expanded our knowledge on the complex relationship between facial attractiveness and various sociological traits including educational aspirations and occupation [19–22]. Recently, dense genotype data have been made available in WLS. These advances make it possible for the first time to identify specific genetic components associated with facial attractiveness and probe its genetic architecture.

We performed a genome-wide association study (GWAS) on 4,383 samples from WLS to identify single-nucleotide polymorphisms (SNPs) associated with facial attractiveness. In addition, sex-stratified analyses suggested distinct genetic architecture between the perception of male and female attractiveness. Integrated analysis of GWAS results and transcriptomic and epigenetic functional annotations also provided mechanistic insights into how genetics may influence facial attractiveness.

## Results

### GWAS identifies genetic loci associated with facial attractiveness

**Fig 1:**
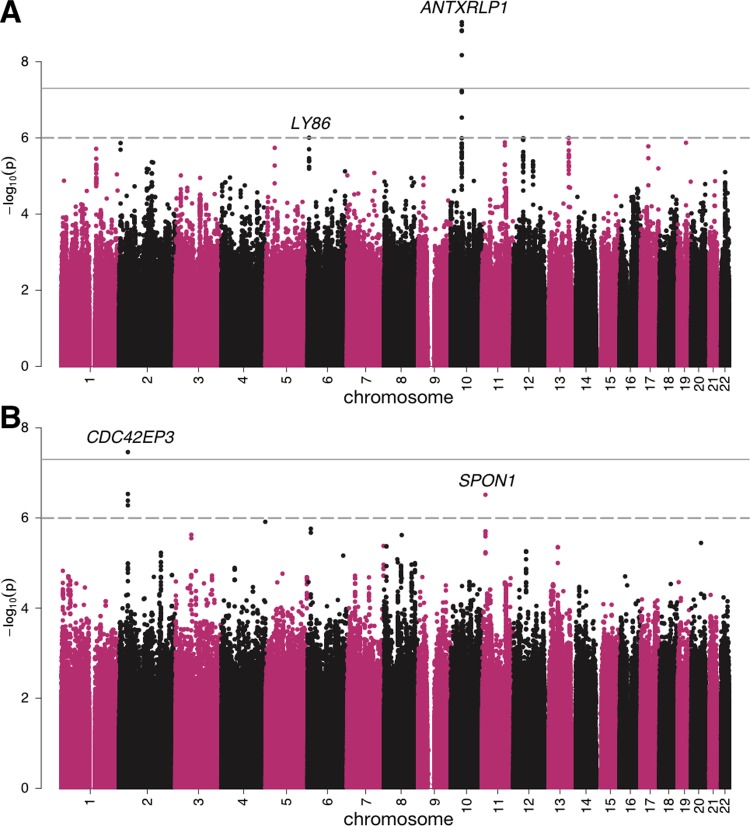
**Manhattan plots for (A) FC-AS and (B) MC-FS analyses.** The horizontal lines denote the genome-wide significance cutoff of 5.0e-8 and a suggestive cutoff of 1.0e-6.

**Fig 2:**
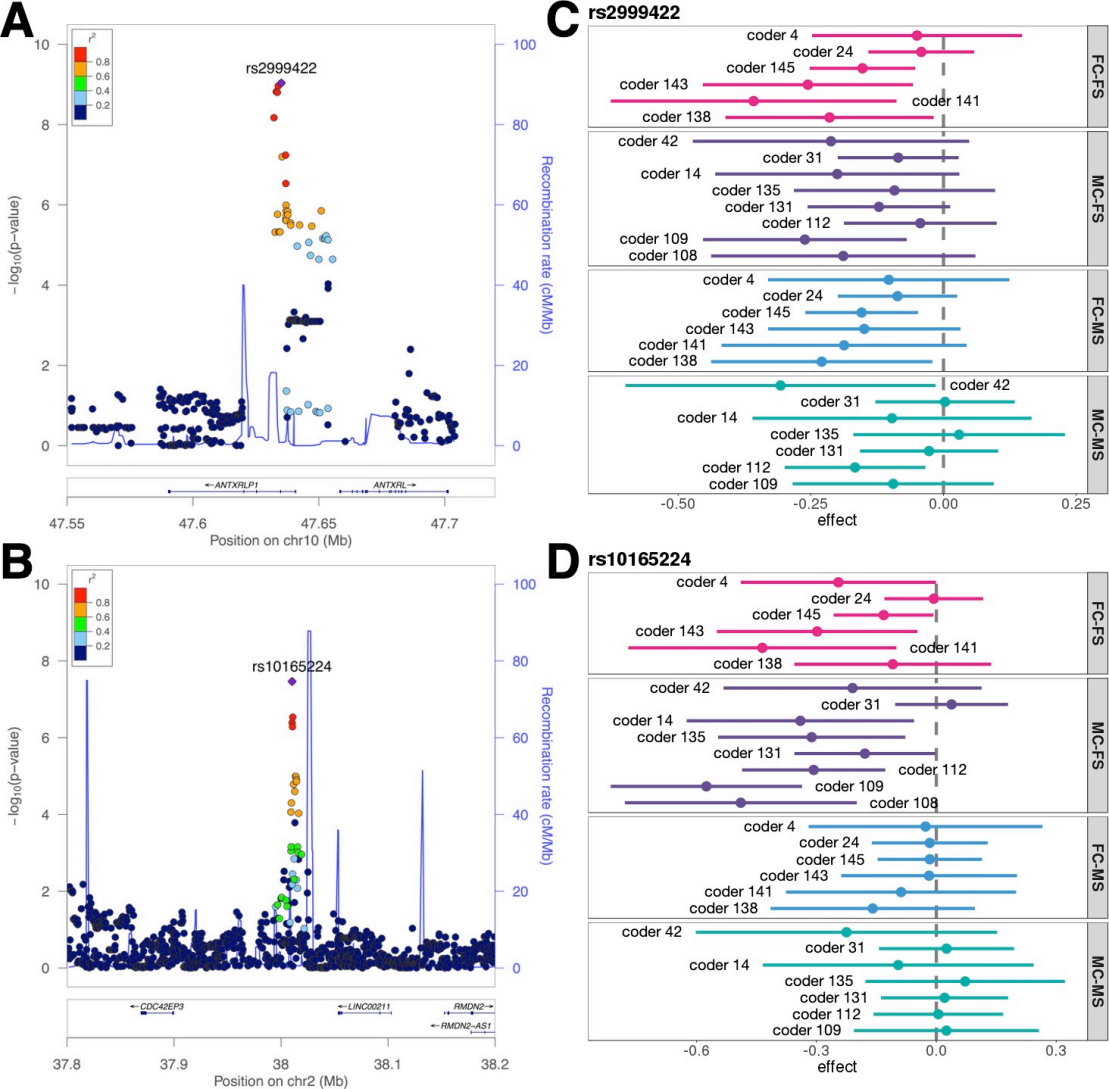
Associations at two genome-wide significant loci. **(A)** Association with FC-AS at 10q11.22. **(B)** Association with MC-FS at 2p22.2. (**C-D**) Associations with each coder’s attractiveness ratings at two genome-wide significant loci. Each interval shows the association with attractiveness score given by one coder. Error bars denote 95% confidence intervals of effect estimates. To maintain sufficient sample size, only coders who rated more than 500 male samples were included in MS studies and coders who rated more than 500 female samples were included in FS studies.

We conducted a GWAS for facial attractiveness on individuals of European ancestry in WLS. After quality control, a total of 3,928 individuals of self-reported European ancestry were included in the discovery stage. Ancestry information was unavailable for a fraction of individuals in WLS. We confirmed the European ancestry for 455 additional individuals using genetic data and used these samples to replicate genome-wide significant findings (**S1 Table**). In 2004 and 2008, each individual’s high-school yearbook photo was independently rated by 12 coders (6 females and 6 males) who were selected from the same birth cohort as the WLS participants. These scores were normalized into two metrics to represent the average attractiveness ratings from female and male coders on each individual (**Methods**). We conducted two separate GWAS on all samples using attractiveness scores given by female and male coders as quantitative traits (**Figs 1A** and **S1 and S2**). These two analyses are referred to as FC-AS (female coders, all samples) and MC-AS (male coders, all samples) throughout the paper. We identified one genome-wide significant locus at 10q11.22 for FC-AS (rs2999422; **Table 1**and **Fig 2A**). Of note, this locus also passed a more stringent study-wise Bonferroni correction adjusted by the total number of traits we analyzed in this study. The leading SNP at this locus showed consistent effect direction in the replication cohort but did not reach statistical significance, possibly due to the limited replication sample size. However, the p-value became more significant in meta-analysis (p = 6.5e-10), showing strengthened statistical evidence. No genome-wide significant loci were detected for MC-AS. Additionally, we identified three loci showing suggestively significant associations (p<1.0e-6) with FC-AS (6p25.1) and MC-AS (20q13.11 and 2q22.1; **S2 Table** and **S3 and S4 Figs**).

**Table 1:** Genome-wide significant loci associated with facial attractiveness.

Trait	Locus	SNP^a^	Genes	Pos (hg19)	Alleles^b^	EAF^c^	Beta_dis_	SE_dis_	P_dis_	Beta_rep_	SE_rep_	P_rep_	Beta_meta_	SE_meta_	P_meta_
FC-AS	10q11.22	rs2999422	*ANTXRLP1*	47635107	G/T	0.43	-0.19	0.03	9.2e-10	-0.09	0.09	0.29	-0.18	0.03	6.5e-10
MC-FS	2p22.2	rs10165224	*CDC42EP3*, *LINC00211*	38010266	G/A	0.20	-0.30	0.05	3.4e-08	-0.16	0.17	0.33	-0.29	0.05	2.3e-08

^a^ The most significant SNP at each locus is listed;

^b^ Reference/effect allele;

^c^ Effect allele frequency

Attractiveness is known to have sex-specific associations with various social factors [23–25]. Thus, we hypothesized that different genetic components may be associated with male and female attractiveness and the genetic architecture may further diverge when comparing the perceptions of male and female coders. We conducted four sex-specific GWAS (**Figs 1B** and **S5 and S6**) based on female coders and female samples (FC-FS), female coders and male samples (FC-MS), male coders and female samples (MC-FS), and male coders and male samples (MC-MS). We identified one additional genome-wide significant locus at 2p22.2 for MC-FS (rs10165224; **Fig 2B**). This locus also showed a consistent effect direction in the replication cohort. Meta-analysis further strengthened the statistical evidence and lowered the p-value (p = 2.3e-8; **Table 1**). Seven loci (i.e. 1q21.3, 5p15.31, 8q24.11, 11p15.2, 12q12, 17p13.3, and 17q11.2) showed suggestive associations in sex-stratified analyses (**S2 Table** and **S7–S10 Figs**). Of note, associated loci identified for FC-AS and MC-AS all showed consistent effects in sex-specific analyses (**S3 Table**). We also formally tested SNP-sex interaction effect for the two genome-wide significant SNPs. With male coded as 1 and female coded as 2, we identified significant interaction effect of sex and rs10165224 for male coders’ ratings (effect = -0.298, p = 6.7e-5). Interaction was nominally significant but much weaker when analyzing ratings of female coders (effect = -0.181, p = 0.015). These results are consistent with effect estimates in the sex-stratified analyses and suggest that rs10165422 has a significantly stronger negative effect on facial attractiveness in females than in males, especially when rated by male coders. Furthermore, we performed an X-chromosome wide association study (XWAS) to investigate sex-specific effects on the X-chromosome. However, no loci reached genome-wide significance in either sex-stratified analysis or meta-analysis (**S4 Table**).

A total of 80 coders participated in the attractiveness study in WLS (**Methods**). To investigate the heterogeneity of identified signals, we performed association analyses using attractiveness scores from each coder separately. In order to maintain statistical power in the association analysis based on ratings from each coder, we focused on coders who rated more than 500 male or female samples in WLS (**S11 Fig**). The genome-wide significant association identified for FC-AS, i.e. rs2999422, showed consistently negative associations with attractiveness scores from all female coders and most male coders (**Fig 2C**). The genome-wide significant locus in MC-FS analysis, rs10165224, also showed consistency–negative associations for all male coders except one (**Fig 2D**). Consistent association patterns were also observed for other identified loci (**S12 Fig**). In addition, we investigated the variability of ratings from different coders (**S13 and S14 Figs**). All tested correlations were statistically significant after Bonferroni correction. These results suggest that attractiveness ratings from different coders were mostly consistent and the associations identified in our analyses were not driven by coder biases. Rather, they represent genetic associations with the consensus of opinions among coders.

### Heritability and selection signatures of facial attractiveness

Consistent with many other complex traits [26], top associations identified in our analyses only explained a small fraction of phenotypic variability. We obtained positive estimates of chip heritability for FC-AS (heritability = 0.109, p = 0.230) and MC-AS (heritability = 0.277, p = 0.036) using genome-wide data [27]. However, we note that standard errors for these estimates were high (0.149 for FC-AS and 0.159 for MC-AS) and the GREML algorithm [27] did not converge in sex-specific analyses, likely due to limited sample size. Analysis based on a different method–GEMMA [28]–yielded similar results (**S5 Table**). Next, we applied linkage disequilibrium (LD) score regression [29] to partition heritability by tissue and cell type. Interestingly, several tissues related to reproduction and hormone production were strongly enriched for heritability of facial attractiveness (**S6 Table**). Despite not reaching statistical significance after correcting for multiple testing, testis was the top tissue for FC-AS (enrichment = 3.9, p = 0.04) and ovary was the most enriched tissue for MC-AS (enrichment = 4.5, p = 0.032), MC-FS (enrichment = 5.7, p = 0.040), and FC-FS (enrichment = 3.0, p = 0.005). Reproductive organs were not highlighted in male-specific analyses (i.e. MC-MS and FC-MS).

**Fig 3:**
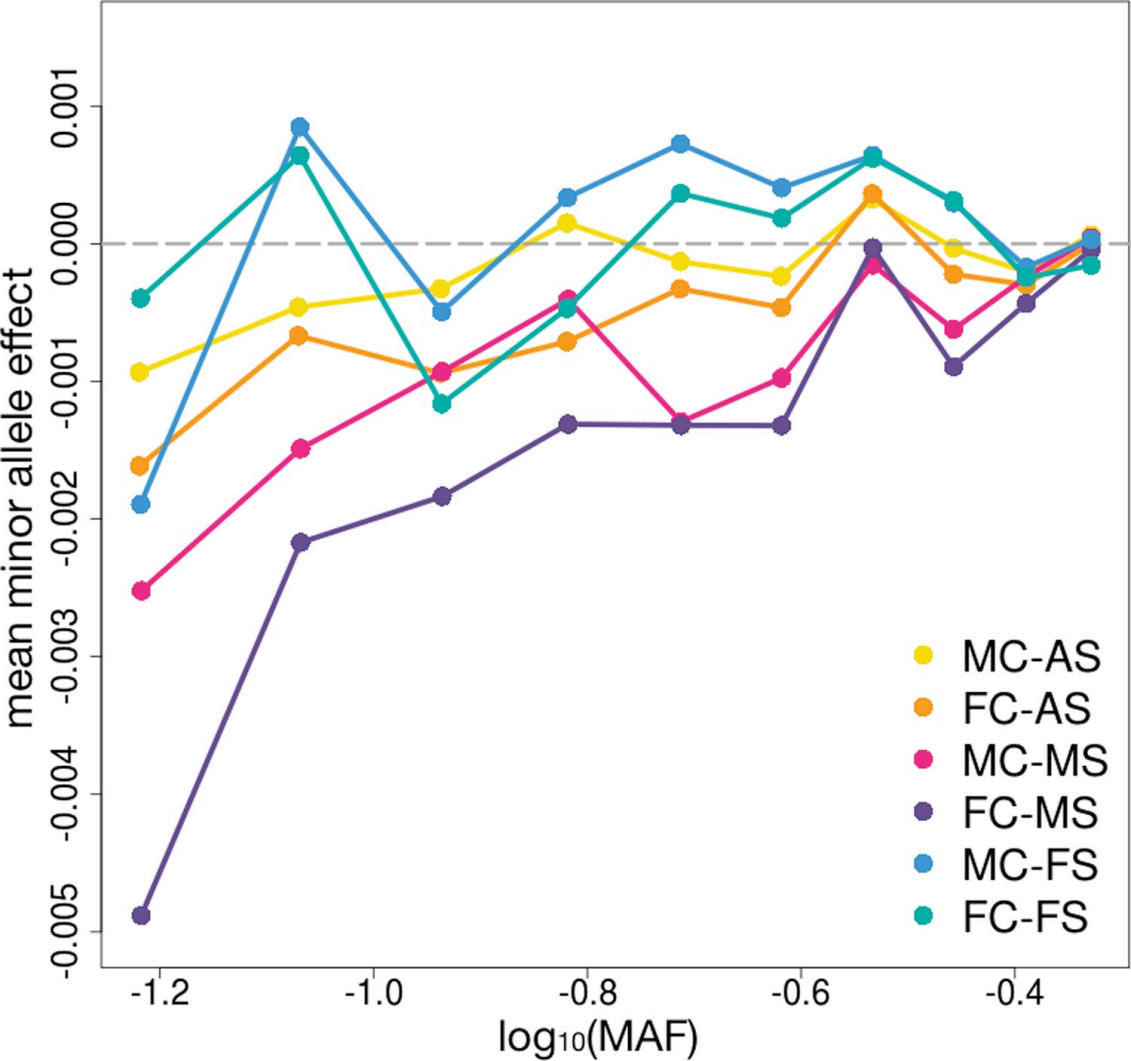
Selection signatures of facial attractiveness. SNPs with MAF between 0.05 and 0.5 were grouped into 10 equally-sized bins based on MAF quantiles. For SNPs in each bin, average MAF was calculated shown on the x-axis, while the average minor allele effects are shown on the y-axis.

Further, following a strategy proposed in [30], we investigated the relationship between minor allele frequencies (MAF) and minor allele effects on facial attractiveness. We grouped SNPs with MAF between 0.05 and 0.5 into 10 equally-sized bins based on MAF quantiles. Interestingly, minor alleles with low frequencies tend to have negative effects on male facial attractiveness (**Fig 3**). The mean minor allele effect on FC-MS from SNPs in the lowest 10% MAF quantile was -0.005, implying very strong statistical evidence for its deviation from zero (p = 7.3e-313; two-sided t-test). SNPs in the highest 10% MAF quantile, however, did not show significantly negative associations (mean effect = -4.1e-5, p = 0.493). This hinted at selection pressure on genetic variants associated with negative male attractiveness. The selection signature in females was not as clear.

### Candidate genes at identified loci

One genome-wide significant locus at 10q11.22 was identified for FC-AS. The leading SNP at this locus, rs2999422, is located in an intron of pseudogene *ANTXRLP1*. The closest protein-coding gene is *ANTXRL* (**Figs 2A** and **S15**). This locus has been previously reported to associate with skin pigmentation [31] and transferrin saturation [32]. The genes closest to the three suggestively significant loci for FC-AS and MC-AS, i.e. *LRP1B*, *PTPRT*, and *LY86* (**S3 and S4 Figs**), have been reported in multiple association studies. Specifically, *LRP1B* is a member of the low-density lipoprotein (LDL) receptor family and is associated with body-mass index (BMI) [33], aging [34], and age at menarche [35]; *PTPRT* is associated with facial morphology [36]; *LY86* is associated with waist-hip ratio [37] and hip circumference [38].

Among the loci identified in sex-stratified analyses, one locus at 2p22.2 reached genome-wide significance for MC-FS (**Fig 2B**). The leading SNP rs10165224 is located in an intergenic region between protein-coding gene *CDC42EP3* and RNA gene *LINC00211*. This locus was known to be associated with height [37, 39]. Genes at the seven suggestively significant loci (**S7–S10 Figs**) were also associated with various traits related to facial features. Both *SPON1* at 11p15.2 and *NXN* at 17p13.3 are associated with facial morphology [36]. *NXN* is also associated with vulvitis and vulva disease (MCIDs: VLV008 and VLV036). Additionally, *EXT1* at 8q24.11 is associated with obesity [40] and *PDZRN4* at 12q12 is associated with BMI [41] and skin pigmentation [31]. Finally, the locus at 1q21.3 contains a large LD block covering multiple genes, among which *ANXA9* is associated with melanoma [42].

A few leading SNPs at identified loci are expression quantitative trait loci (eQTL) for nearby genes (**S7 Table**). The genome-wide significant SNP at 10q11.22 for FC-AS, rs2999422, is an eQTL for *ANTXRL* across various tissues (minimum p = 1.1e-8); rs17746363 is an eQTL for *MED30* in skeletal muscle (p = 2.5e-6); rs2074151 and rs6587551 are eQTL in thyroid for *RAB11FIP4* (p = 1.2e-5) and *CTSS* (p = 5.6e-26), respectively. To systematically utilize multi-tissue eQTL data and better quantify associations at the gene level, we performed cross-tissue transcriptome-wide association analyses for six facial attractiveness traits using the UTMOST method (**Methods**; **S16 Fig**) [43]. We identified four significant gene-level associations after correcting for multiple testing: *SYT15* at 10q11.22 for FC-AS (p = 1.0e-6), *CTSS* at 1q21.3 for FC-MS (p = 9.6e-7), *RPL22* at 1p36.31 for MC-FS (p = 1.5e-7), and *ATAD5* at 17q11.2 for MC-MS (p = 8.9e-7). *SYT15* is 700kb upstream of *ANTXRL*, the genome-wide significant locus for FC-AS. *CTSS* is located at a suggestively significant locus for FC-MS and is associated with BMI [44]. *ATAD5* is 700kb upstream of the suggestively significant locus for MC-MS and is known to associate with many complex traits including height, waist circumference, hip circumference, and BMI [45–47]. *RPL22* is a novel association. All gene-level associations for six attractiveness traits are summarized in **S8 Table**.

### Relationship between facial attractiveness and other complex traits

Next, we investigated the relationship between facial attractiveness and various related human traits. First, we tested the enrichment for associations with six dermatological traits related to skin and hair pigmentation (**S9 Table**) among top SNPs identified in the attractiveness GWAS (**Methods**). Overall, SNPs associated with female coders’ ratings were enriched for associations with hair pigmentation while SNPs for male coders’ attractiveness ratings were more strongly enriched for associations with skin pigmentation. Ten enrichment results reached statistical significance after Bonferroni correction (**S10 Table**). Specifically, SNPs associated with FC-AS were significantly enriched for associations with multiple hair color traits, i.e. blonde (p = 5.3e-05), light brown (p = 4.2e-4), dark brown (p = 2.8e-4), and red (p = 3.2e-4). Top SNPs for MC-AS were only significantly enriched for skin pigmentation (p = 3.8e-4). A similar preferential distinction between male and female coders was also observed in sex-stratified analyses. Top SNPs for FC-FS were enriched for associations with dark brown hair (p = 8.2e-4) and top SNPs for FC-MS were enriched for associations with blonde (p = 4.4e-5), red (p = 4.2e-4), and black hair (p = 1.7e-5). In contrast, top SNPs for MC-MS were enriched only for associations with skin pigmentation (p = 3.2e-4). No significant enrichment was observed for MC-FS.

**Fig 4:**
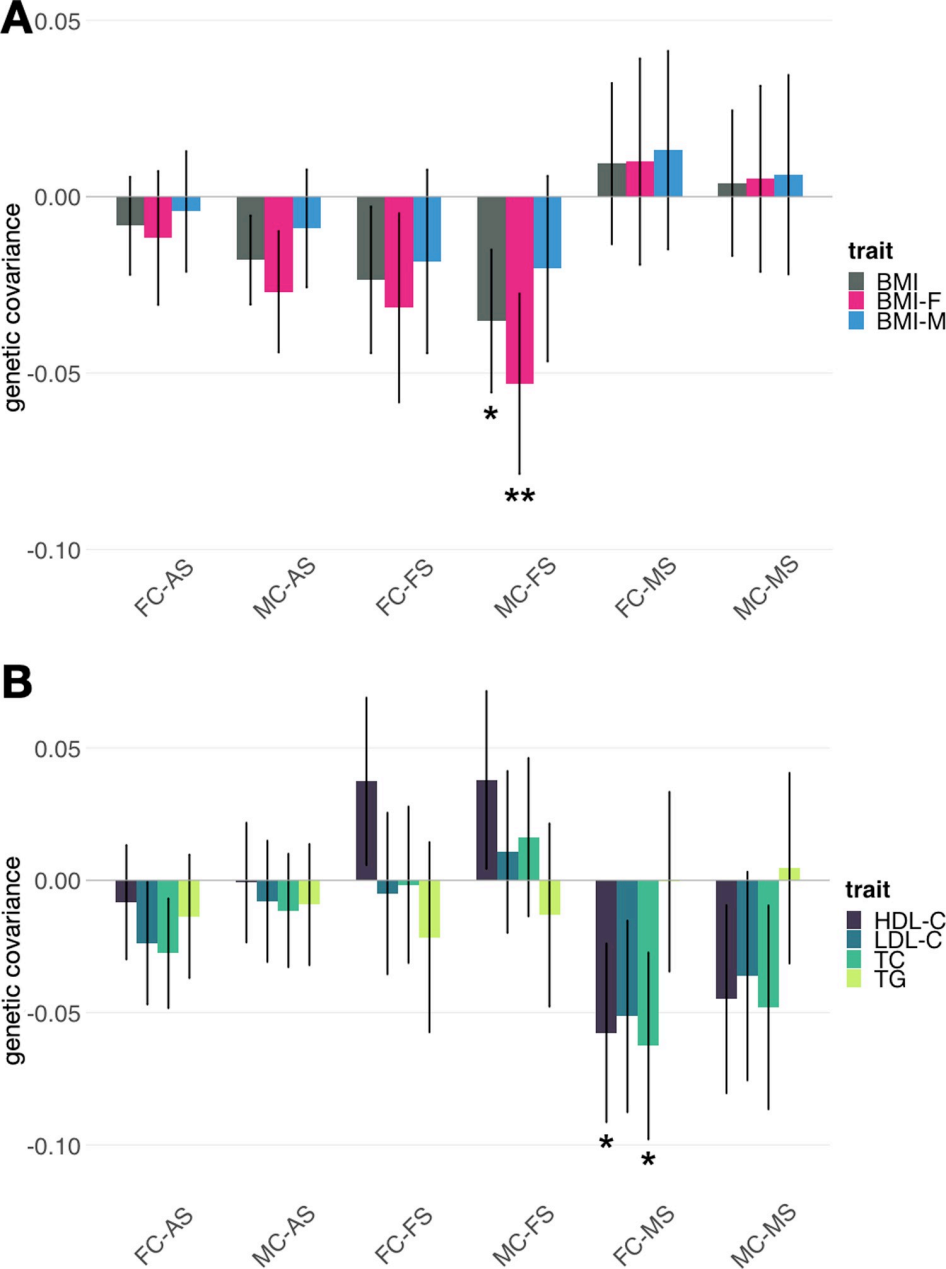
**Genetic covariance between facial attractiveness and (A) BMI traits, (B) lipid traits.** Intervals show the 95% confidence intervals of covariance estimates. Genetic covariance with fdr < 0.1 are marked by one asterisk and the covariance with p-value < 1e-4 is marked by two asterisks.

To further reveal the polygenic relationship between facial attractiveness and other complex traits, we estimated genetic covariance between facial attractiveness and 50 complex traits with publicly accessible GWAS summary statistics which covered a spectrum of social, psychiatric, anthropometric, metabolic, and reproductive phenotypes (**S11 Table**). Results for all 300 pairs of genetic covariance are summarized in **S12 Table**. One pair of traits–female BMI (BMI-F) and MC-FS, showed a strong and negative correlation, and the p-value achieved Bonferroni-corrected significance (covariance = -0.053, p = 4.7e-5). Additionally, three other pairs of traits did not reach statistical significance but showed genetic covariance with Benjamini-Hochberg false discovery rate (fdr) below 0.1, including BMI and MC-FS (covariance = -0.035; p = 6.4e-4), high-density lipoprotein cholesterol (HDL-C) and FC-MS (covariance = -0.058; p = 8.2e-4), and total cholesterol (TC) and FC-MS (covariance = -0.063; p = 5.1e-4). Interestingly, female attractiveness traits were negatively correlated with all three BMI traits, and the correlation signal was the strongest when attractiveness was rated by male coders (i.e. MC-FS) and the BMI analysis was specific to females (**Fig 4A**). However, such a relationship was completely absent between male attractiveness and BMI. In fact, both FC-MS and MC-MS were positively correlated with BMI traits although the p-values were non-significant. In contrast, genetic covariance between attractiveness and lipid traits was specific to male samples, especially the FC-MS analysis in which female coders’ scores were analyzed (**Fig 4B**).

Furthermore, we explored if the strong genetic covariance of MC-FS with BMI-F could be explained by a causal relationship between these traits. We performed robust Mendelian randomization (MR) to infer causality (**Methods**). We identified a negative effect (effect = -1.05, SE = 0.62) from BMI-F to MC-FS with p = 0.099. Our results hinted at a causal effect between BMI-F and MC-FS but the validity of the relationship requires future investigation using larger samples.

## Discussion

Despite tremendous interests from both academia and industry, the genetic basis of facial attractiveness is poorly understood, partly due to the scarcity of well-phenotyped facial attractiveness in large-scale cohorts with genetic information. Carefully-measured human facial attractiveness, in conjunction with dense genotype data in WLS, made it possible to identify specific genetic components for facial attractiveness. In this paper, we conducted a GWAS to identify DNA variants associated with human facial attractiveness. We identified two genome-wide significant loci on 10q11.22 and 2p22.2 and highlighted several genes via eQTL analysis and transcriptome-wide association analysis. Human tissues involved in reproduction and hormone production were implicated in heritability enrichment analysis. Additionally, we identified evidence for shared genetics between attractiveness and other complex traits. Top SNPs for attractiveness were enriched for associations with dermatological traits related to skin and hair pigmentation. Via a genome-wide genetic covariance estimation approach, we identified strong evidence for shared genetic architecture of facial attractiveness with BMI and lipid traits. Of note, sex-specific genetic architecture of facial attractiveness was a recurrent pattern observed in almost all our analyses. The loci that reached significance in analyses based on all samples showed consistent effects between males and females, but sex-specific analyses revealed a list of new loci. The leading SNP at genome-wide significant locus 2p22.2 showed a significant interaction effect with sex. In multi-trait analyses, SNPs associated with female coders’ attractiveness ratings were enriched for associations with hair color while top SNPs for male coders’ ratings were enriched for associations with skin pigmentation. Additionally, only female attractiveness (especially MC-FS) showed strong and negative genetic correlation with BMI while male attractiveness was more strongly correlated with blood cholesterol levels which are known to be involved in the synthesis of testosterone and other steroid hormones [48]. Finally, variants and genes were identified for both male and female attractiveness but the selection pressure on negative associations of male attractiveness seemed to be particularly strong. These results not only provided fundamental new insights into the genetic basis of facial attractiveness, but also revealed the complex relationship between attractiveness and a variety of human traits.

This study was not without limitations. First, although WLS provided a great opportunity to study the genetics of facial attractiveness, the sample size was moderate and we did not find an external cohort to replicate our association findings due to the uniqueness of this phenotype. Although heritability of facial attractiveness has been demonstrated in twin studies [17, 18], we were unable to obtain statistically significant results on chip heritability in our study. Due to weak effect sizes, extreme multiple testing, and ubiquitous confounding, external replication and validation are critical steps in studies of complex trait genetics. In our analysis, we used 455 samples with genetically confirmed European ancestry in WLS to replicate genome-wide significant findings and performed a variety of analyses to assess the heterogeneity of identified associations, including comparing association signals between males and females as well as across different coders. The effect directions of both genome-wide significant SNPs were consistent between the discovery and replication stages and the p-values became more significant in the meta-analysis. Still, spurious associations remain a possibility and the validity of our findings needs to be further investigated using independent samples. Second, attractiveness measurements in WLS were based on high-school yearbook photos. Although it is a common practice to use photos as the basis of attractiveness measurements [11, 18, 49], our phenotyping approach does not cover every aspect of attractiveness and the results need to be interpreted with caution. In our study, each photo was rated by 12 different coders and the rating scores were consistent across coders. These results suggest the robustness of the phenotypic measurements in our study, but many questions remain unanswered. What are the roles of age, physical body shape, facial expression, and make-up in the perception of attractiveness? What is the impact of assortative mating on the genetics of attractiveness [50]? And what is the shared and distinct genetics between attractiveness and closely related facial phenotypes such as symmetry, averageness, and sexually dimorphic features [14]? These are just a handful of questions beyond the scope of this study. We also note that since each yearbook photo in WLS was rated by 6 female and 6 male coders, we were able to obtain robust phenotypic measurements based on male and female coders separately. This stratification proved critical for some analyses we conducted in this study. However, for future replication using other cohorts without sufficient numbers of male and female-specific ratings, it may be necessary to conduct additional GWAS by combining all coders’ ratings. Additionally, we note that both the raters and the people being rated were from one state that was racially and ethnically quite homogeneous and we only included samples with European ancestry in this study. Further, the yearbook photos in WLS were collected more than sixty years ago. It is unclear how well our results can be generalized to other populations, age groups, and generations. If the same high school yearbook photos were to be rated for facial attractiveness by a more ethnically or racially diverse set of raters, and if the findings were to be replicated, then the inference regarding genetic association of attractiveness would be strengthened. Nevertheless, this study was a successful attempt to pin down genetic components of human facial attractiveness. Many of these unanswered questions will be exciting directions to explore in the future. We have little doubt that robust and comprehensive phenotypic measurements, coupled with larger sample sizes from diverse populations, will further advance our understanding of this interesting human trait.

## Methods

### WLS data details

WLS is a longitudinal study of a 1/3 random sample of over ten thousand Wisconsin high school graduates in 1957. Facial attractiveness in WLS was measured based on each individual’s 1957 high school yearbook photo by 12 coders (six females and six males) selected from the same cohort in 2004 and 2008. The subjects in the photos were of the same age and the photos had the same yearbook format. In total, 80 coders were involved in the study and not all photos were rated by the same group of coders. An 11-point rating scale was used to quantify attractiveness. End-points of rating were labeled as “not at all attractive” and “extremely attractive” for 1 and 11, respectively. In this study, we used normalized average ratings from female coders and normalized average ratings from male coders as two quantitative traits for facial attractiveness. Normalization was performed in a prevailing fashion as subtracting mean and then dividing by standard deviation.

Genetic data were obtained from saliva samples in the years 2006 and 2007 using Oragene kits and a mail-back protocol. All participants provided informed consent under a protocol approved by the Institutional Review Board of the University of Wisconsin-Madison. Genotyping was conducted using the Illumina Human Omni Express Bead Chip. 713,014 SNPs were genotyped. The quality control process was previously conducted for a published GWAS on educational attainment [51]. We used genotype data imputed against the Haplotype Reference Consortium (HRC) panel. Individuals were removed if they met one of the following criteria: 1) genotype missingness rate > 0.05; 2) surveyed sex did not match genetic sex; 3) surveyed relatedness did not match genetic relatedness; 4) the individual was an outlier in respect of heterozygosity or homozygosity (F statistic > 0.03 or < -0.03); 5) the individual was an ancestral outlier–we iteratively dropped individuals with nearest neighbor z-score < -5 until no more individuals with a z-score < -5 remain. In addition to these quality control criteria, we only included individuals with available attractiveness ratings, self-reported European ancestry, and birthday between 1937–1940 in the study. SNPs were removed if: 1) call rate < 0.95; 2) Hardy-Weinberg exact test p-value < 1.0e-5; 3) minor allele frequency < 0.01; or 4) imputation quality score < 0.8. After quality control, 7,251,583 autosomal SNPs and 3,928 individuals remained in the discovery stage.

### GWAS analysis

In the analysis using all samples (i.e. MC-AS and FC-AS), we applied linear mixed model (LMM) implemented in the GCTA software [52] to perform association analysis while correcting for relatedness among samples. In addition, sex, round of coding (i.e. was attractiveness rated in 2004 or 2008), dummy variables for birth year were included in the model as covariates. In sex-stratified association analyses, we applied linear regression instead of LMM due to the reduction in sample size and the consequent non-convergence of the restricted maximum likelihood algorithm and add the first two principal components into covariates. We used the prevailing p-value cutoff 5e-8 to claim genome-wide significance and 8.3e-9 as the study-wise significance cutoff to further adjust for 6 traits we analyzed. In addition, we used 1e-6 as a suggestive significance cutoff. WLS data were collected on high school graduates of the same year and distant cousins may be involved due to the study design. To adjust for family structure in linear regression analysis, we kept only one individual in each pair of samples with relatedness coefficient greater than 0.05. Relatedness coefficients among samples were estimated using PLINK [53]. After these additional quality control steps, 1,792 male samples and 2,062 female samples remained in sex-stratified analyses, PLINK was used to perform association analysis with sex, round of coding, birth year, and the first two principal components (PCs) included as covariates. We also used PLINK to test the interaction between genome-wide significant SNPs and sex using all samples. Males were coded as 1 and females were coded as 2. Significance was determined by a Bonferroni-corrected p-value cutoff (i.e. 0.05/4) which adjusted for two SNPs and two traits (i.e. attractiveness ratings based on male and female coders).

### Replication

We replicated genome-wide significant findings from the discovery stage using WLS samples who did not report ancestry information but had genetically confirmed European ancestry. Scatter plot based on top two PCs for WLS and 1000 Genome samples [54] is shown in **S17 Fig**. Quality control procedure in the replication dataset is the same with that in the discovery stage. 455 individuals passed quality control and were used to replicate the association of rs2999422 with FC-AS, and 213 female samples were used to replicate the sex-stratified association between rs10165224 and MC-FS. We used linear regression implemented in PLINK to perform association analysis. Inverse variance-weighted method was applied to meta-analyze results from the discovery and replication stages.

### XWAS

SNPs on the X-chromosome were imputed against HRC panel using the Michigan Imputation Server [55]. Variants were removed if 1) missing call rates > 0.1; 2) MAF < 0.005; 3) significant deviation from Hardy-Weinberg equilibrium in women (p<1e-6); 4) imputation quality score < 0.8. 5) located in the pseudo-autosomal regions (PARs), or 6) MAF between males and females was significant (p<0.001). Additionally, individuals were removed if their reported sex did not match the heterozygosity rates observed on chromosome X. After these quality control steps, 156,615 SNPs and 3,921 samples (2,102 females and 1,819 males) were included in our analyses. We used XWAS software [56, 57] to perform sex-stratified tests on X-chromosome. We added the first two PCs calculated from autosomes as covariates to adjust for population stratification. Round of coding, birth year were also included in the model as covariates. Fisher’s method and Stouffer's method implemented in XWAS were used to meta-analyze male and female samples (i.e. FC-AS and MC-AS).

### eQTL data and transcriptome-wide association analysis

Multi-tissue gene expression and eQTL data were acquired from data portal of the Genotype-Tissue Expression (GTEx) project (https://www.gtexportal.org). We applied UTMOST [43] to perform cross-tissue transcriptome-wide association analysis for six facial attractiveness traits. We used cross-tissue gene expression imputation models trained from 44 tissues in GTEx [58]. Gene-level association meta-analysis was performed using generalized Berk-Jones test [59] implemented in UTMOST software. Statistical significance was determined using a Bonferroni corrected p-value cutoff 3.2e-6.

### Heritability and multi-trait analysis

The GREML method implemented in GCTA software was used to estimate heritability of facial attractiveness [60, 61]. We also used GEMMA as an alternative approach to validate the results [28]. Sex, round of coding, and dummy variables for birth year were included as covariates. We applied stratified LD score regression [62] implemented in the LDSC software to perform heritability enrichment analysis and identify biologically relevant tissues for facial attractiveness. Tissues with sample sizes greater than 100 in GTEx were included in the analyses. In sex-stratified analyses, non-existent tissues (e.g. testis for females and ovary for males) were removed from the analyses. For each tissue, functional annotation was defined as regions near highly expressed genes (within 50,000 bp up- or downstream). We used median transcripts per million (TPM) as the criterion to select top 10% highly expressed genes. We then estimated partitioned heritability using functional annotation for each tissue while including 53 baseline annotations in the model. P-values were calculated using z-scores of regression coefficient as previously suggested [62].

GWAS summary statistics for six dermatological traits in the UK biobank were downloaded from GWAS atlas (http://atlas.ctglab.nl). After clumping the data using an LD cutoff of 0.1, we tested if top SNPs associated with each attractiveness trait (p < 0.05 in attractiveness GWAS) were enriched for SNPs associated with skin and hair pigmentation (p < 0.05 in the corresponding GWAS). We used hypergeometric test to assess enrichment and a Bonferroni-corrected p-value cutoff to claim statistical significance (p<0.05/36 = 0.0014). We used the GNOVA method [63] to estimate genetic covariance between complex traits. Association statistics of six facial attractiveness traits were jointly analyzed with publicly accessible GWAS summary statistics for 50 complex traits (**S11 Table**). Since samples in WLS were not used in those 50 published datasets, uncorrected genetic covariance estimates were reported in our analyses. Additionally, due to numerically unstable estimates for heritability, we report genetic covariance instead of genetic correlation throughout the paper.

We used MR-Egger [64] approach implemented in ‘MendelianRadomization’ R package to perform causal inference between complex traits. We selected instrumental SNP variables by applying a LD cutoff of 0.05 and a p-value cutoff of 1.0e-9. Based on these criteria, 31 top SNPs for BMI were included in our analysis.

### Other bioinformatics tools

Manhattan plots and QQ plots were generated using ‘qqman’ package in R [65]. Locus plots for GWAS loci were generated using LocusZoom [66].

### Data availability

Genotype data from WLS are available to the research community through the dbGaP controlled-access repository at accession phs001157.v1.p1. Phenotypic data in WLS can be accessed via the WLS data portal (https://www.ssc.wisc.edu/wlsresearch). Summary statistics for facial attractiveness are available at (ftp://ftp.biostat.wisc.edu/pub/lu_group/Projects/Attractiveness).

## Supporting information

S1 Fig Manhattan plot for MC-AS. The horizontal lines denote the genome-wide significance cutoff of 5.0e-8 and a suggestive cutoff of 1.0e-6, respectively. The closest gene at each suggestively significant locus was labeled.
(PNG)

S2 Fig QQ plots for (A) FC-AS and (B) MC-AS.
(PNG)

S3 Fig A suggestively significant locus (6p25.1) associated with FC-AS. (PNG)

S4 Fig Suggestively significant loci associated with MC-AS. **(A)** Associations at locus 2q22.1; **(B)** Associations at locus 20q13.11.
(PNG)

S5 Fig Manhattan plots for (A) FC-FS, (B) FC-MS, and (C) MC-MS. The horizontal lines denote the genome-wide significance cutoff of 5.0e-8 and a suggestive cutoff of 1.0e-6, respectively. The closest gene at each suggestively significant locus was labeled.
(PNG)

S6 Fig QQ plots for (A) FC-FS, (B) MC-FS, (C) FC-MS, and (D) MC-MS.
(PNG)

S7 Fig Suggestively significant loci associated with FC-FS. **(A)** Associations at locus 17p13.3; **(B)** Associations at locus 8q24.11.
(PNG)

S8 Fig A suggestively significant locus (11p15.2) for MC-FS. (PNG)

S9 Fig Suggestively significant loci associated with FC-MS. **(A)** Associations at locus 1q21.3; **(B)** Associations at locus 5p15.31; **(C)** Associations at locus 12q12.
(PNG)

S10 Fig A suggestively significant locus (17q11.2) for MC-MS. (PNG)

S11 Fig Number of yearbook photos rated by each coder. Coders who rated more than 500 male or female samples’ photos were included in association analyses based on single coders’ scoring. Coders with too few sample size were omitted from this figure.
(PNG)

S12 Fig Attractiveness association signals at identified loci across different coders. (PNG)

S13 Fig Heatmap of correlations among ratings of different coders. Coders who rated more than 500 photos in 2008 were analyzed. Color indicates different level of correlation. All correlations shown in the figure were statistically significant after Bonferroni correction.
(PNG)

S14 Fig Histograms of variance and interval sizes (max-min) of attractiveness ratings. (PNG)

S15 Fig Multi-tissue gene expression profile of (A) ANTXRL and (B) ANTXRLP1 in GTEx. Blue and red boxes represent data based on male and female samples, respectively. Both ANTXRL and ANTXRLP1 have higher expression in testis than in other tissues, but the absolute expression values are low.
(PNG)

S16 Fig Manhattan plots for gene-level associations in cross-tissue transcriptome-wide association analyses. The horizontal line denotes the Bonferroni-corrected significance threshold.
(PNG)

S17 Fig Principal components plot for WLS and 1000 Genomes samples. Deep blue circles represent individuals with European ancestry in 1000 Genome (EUR), orange and light blue circles represent WLS samples with self-reported European ancestry (labeled as WLS) and missing but genetically confirmed ancestry information (labeled as REP).
(PNG)

S1 Table Demographic information of study samples. (XLSX)

S2 Table Suggestively significant loci for facial attractiveness. (XLSX)

S3 Table Sex-specific effects of 4 loci identified for FC-AS and MC-AS. (XLSX)

S4 Table Results of X-chromosome wide association analysis. For traits FC-AS and MC-AS, associated loci with p<1e-4 in meta-analysis are shown in the table. For FC-FS, MC-FS, MC-MS, and FC-MS, loci with p<1e-4 in sex-stratified analyses are shown.
(XLSX)

S5 Table Heritability estimates based on GEMMA. The GEMMA algorithm did not converge for MC-MS, FC-MS, and MC-FS.
(XLSX)

S6 Table Tissue-specific heritability enrichment for attractiveness traits. Top five tissues with the highest z-scores were listed for each tissue. MC-MS was not included in the table because no tissue had positive z-scores in our analysis.
(XLSX)

S7 Table eQTL effects of leading SNPs associated with facial attractiveness. (XLSX)

S8 Table Gene-level associations for facial attractiveness in cross-tissue transcriptome-wide association analyses. (XLSX)

S9 Table Information about the six dermatological traits in the UK Biobank. (XLSX)

S10 Table Enrichment for associations with six dermatological traits among attractiveness-associated SNPs. (XLSX)

S11 Table 50 complex traits covering a variety of complex human phenotypes with publicly accessible GWAS summary statistics. (PDF)

S12 Table Genetic covariance between 6 facial attractiveness traits and 50 complex human traits. (XLSX)
