# Supplementary figures and images for: Genome-wide association study reveals sex-specific genetic architecture of facial attractiveness
Source: PLoS Genet. 2019 Apr 4;15(4):e1007973. doi: 10.1371/journal.pgen.1007973 (PMC6448826; doi:10.1371/journal.pgen.1007973)

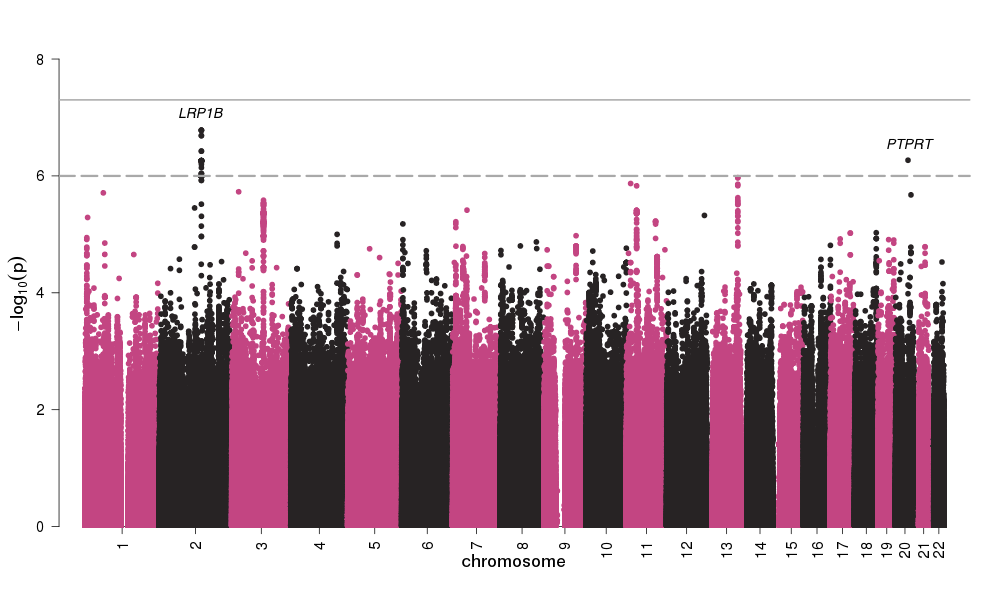

Supplement: S1 Fig — The horizontal lines denote the genome-wide significance cutoff of 5.0e-8 and a suggestive cutoff of 1.0e-6, respectively. The closest gene at each suggestively significant locus was labeled. (PNG) [file pgen.1007973.s001.png]

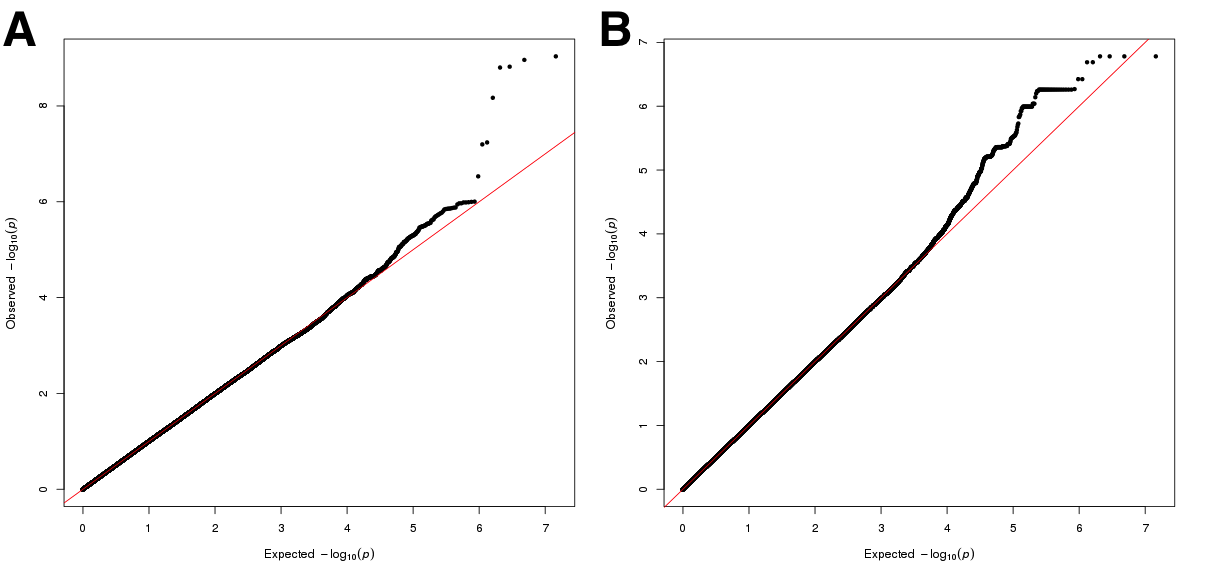

Supplement: S2 Fig — QQ plots for (A) FC-AS and (B) MC-AS. (PNG) [file pgen.1007973.s002.png]

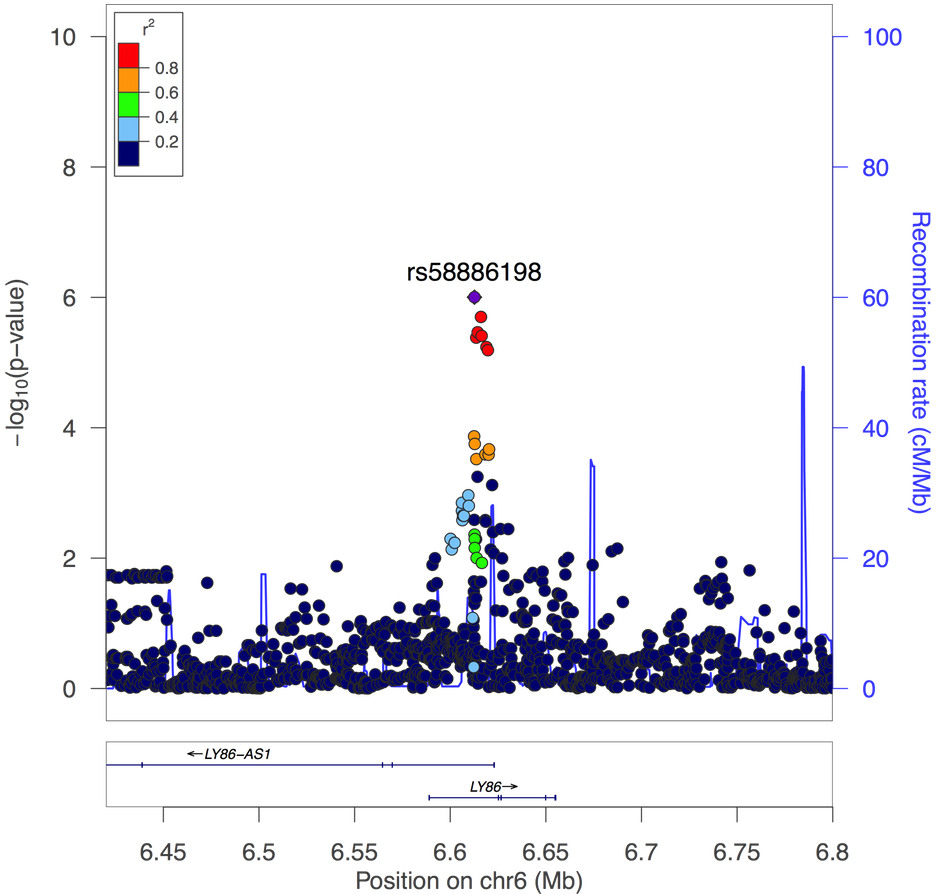

Supplement: S3 Fig — (PNG) [file pgen.1007973.s003.png]

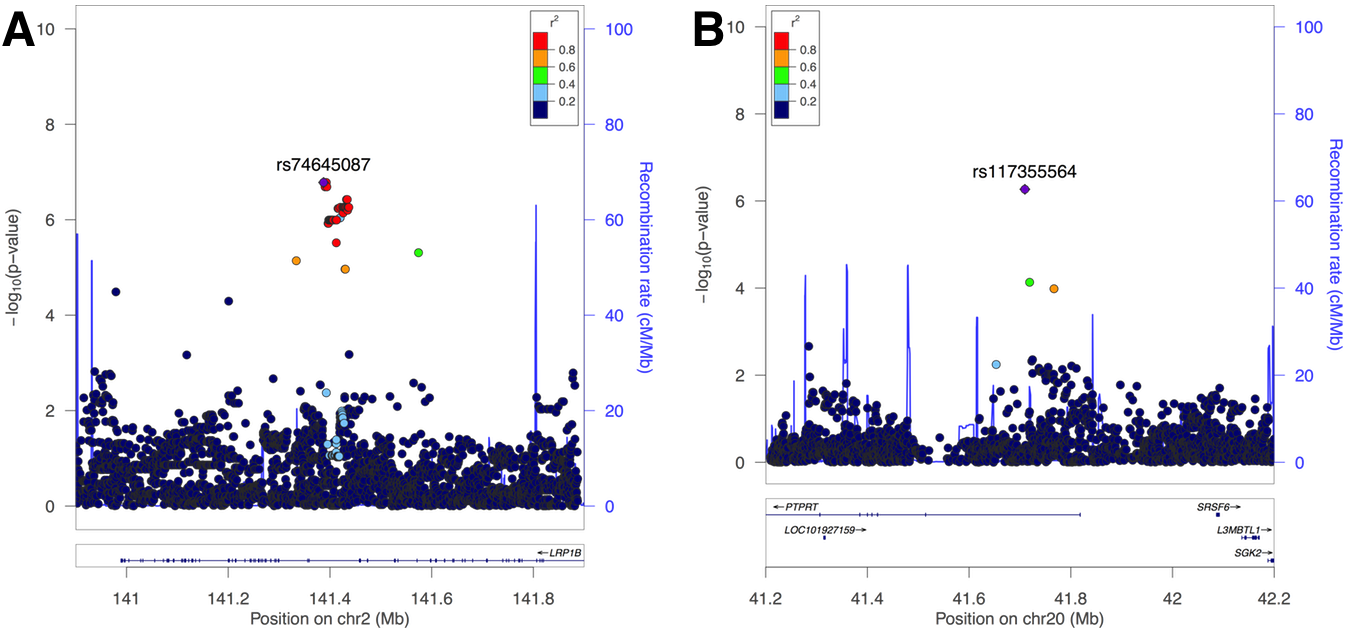

Supplement: S4 Fig — (A) Associations at locus 2q22.1; (B) Associations at locus 20q13.11. (PNG) [file pgen.1007973.s004.png]

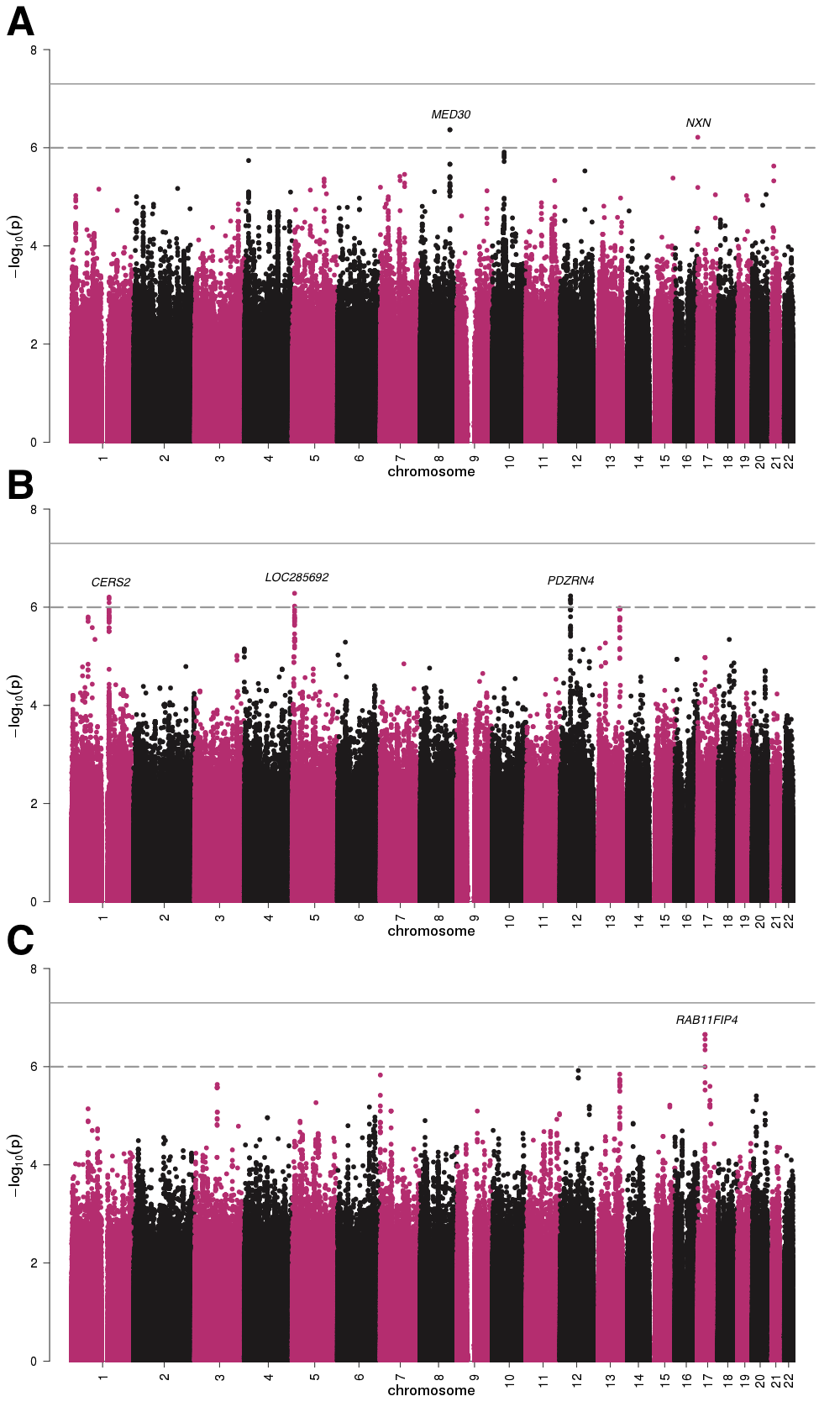

Supplement: S5 Fig — Manhattan plots for (A) FC-FS, (B) FC-MS, and (C) MC-MS. The horizontal lines denote the genome-wide significance cutoff of 5.0e-8 and a suggestive cutoff of 1.0e-6, respectively. The closest gene at each suggestively significant locus was labeled. (PNG) [file pgen.1007973.s005.png]

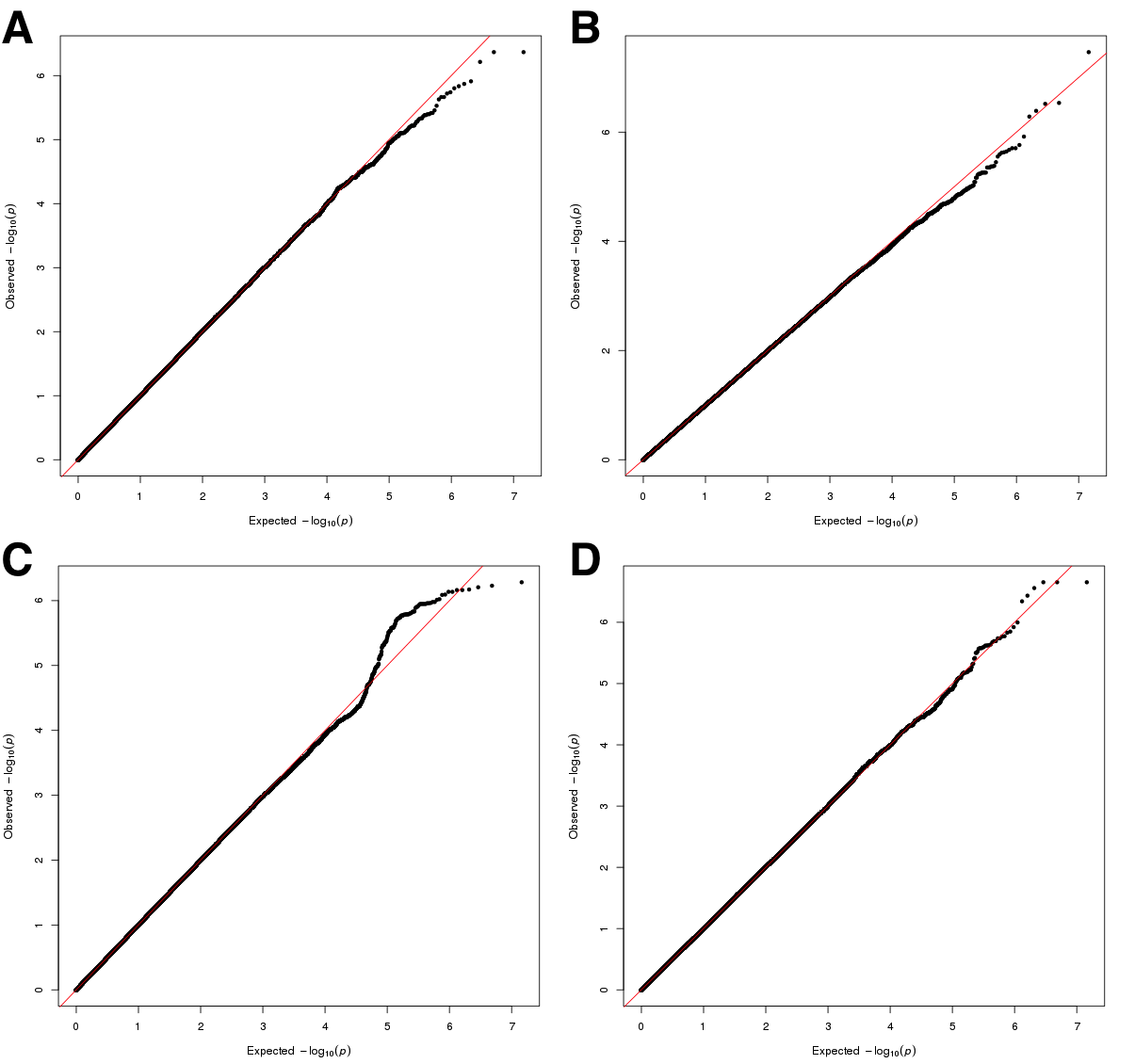

Supplement: S6 Fig — QQ plots for (A) FC-FS, (B) MC-FS, (C) FC-MS, and (D) MC-MS. (PNG) [file pgen.1007973.s006.png]

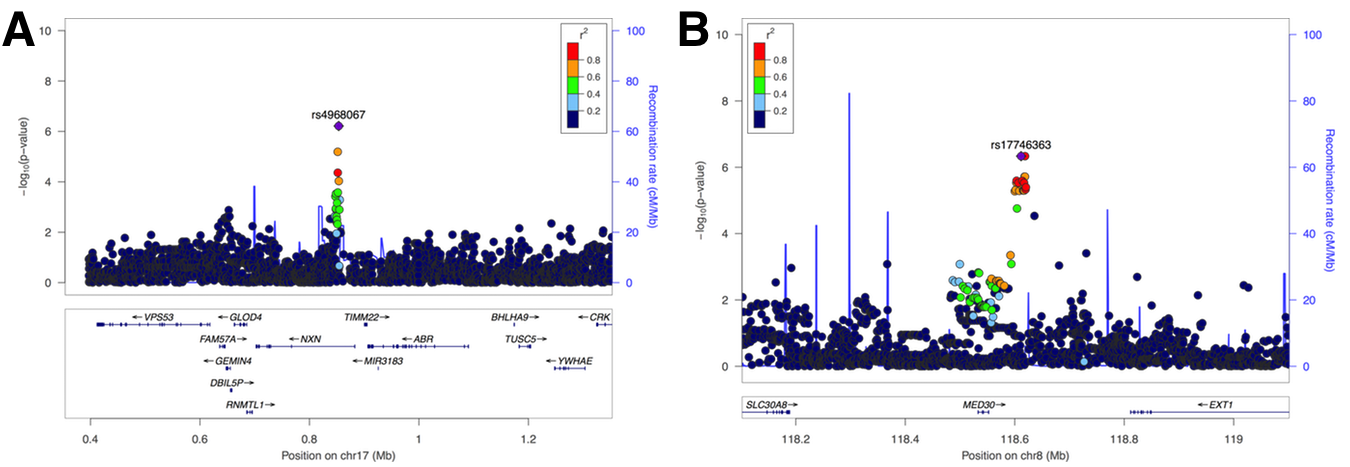

Supplement: S7 Fig — (A) Associations at locus 17p13.3; (B) Associations at locus 8q24.11. (PNG) [file pgen.1007973.s007.png]

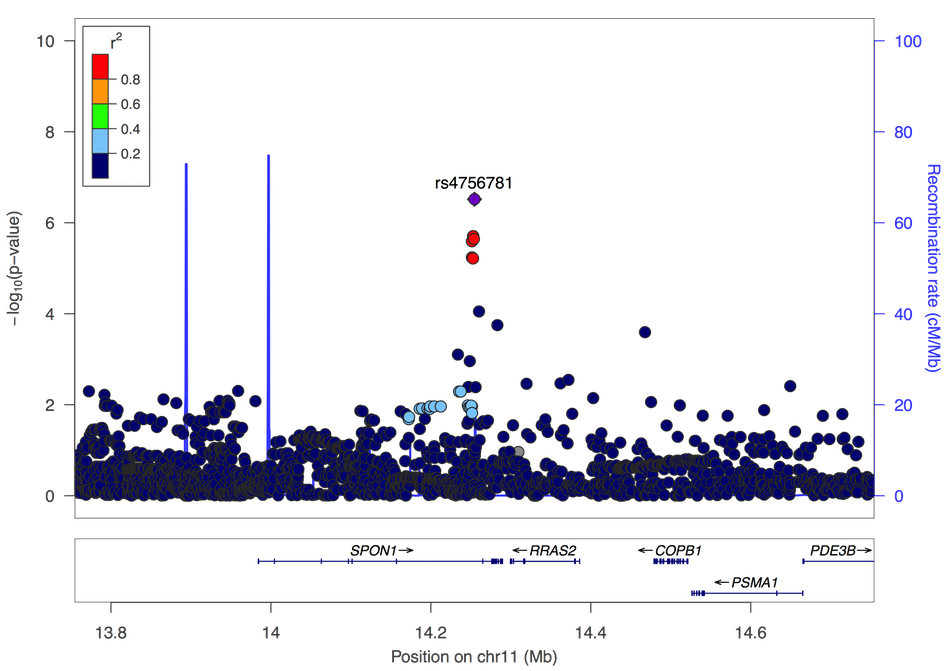

Supplement: S8 Fig — (PNG) [file pgen.1007973.s008.png]

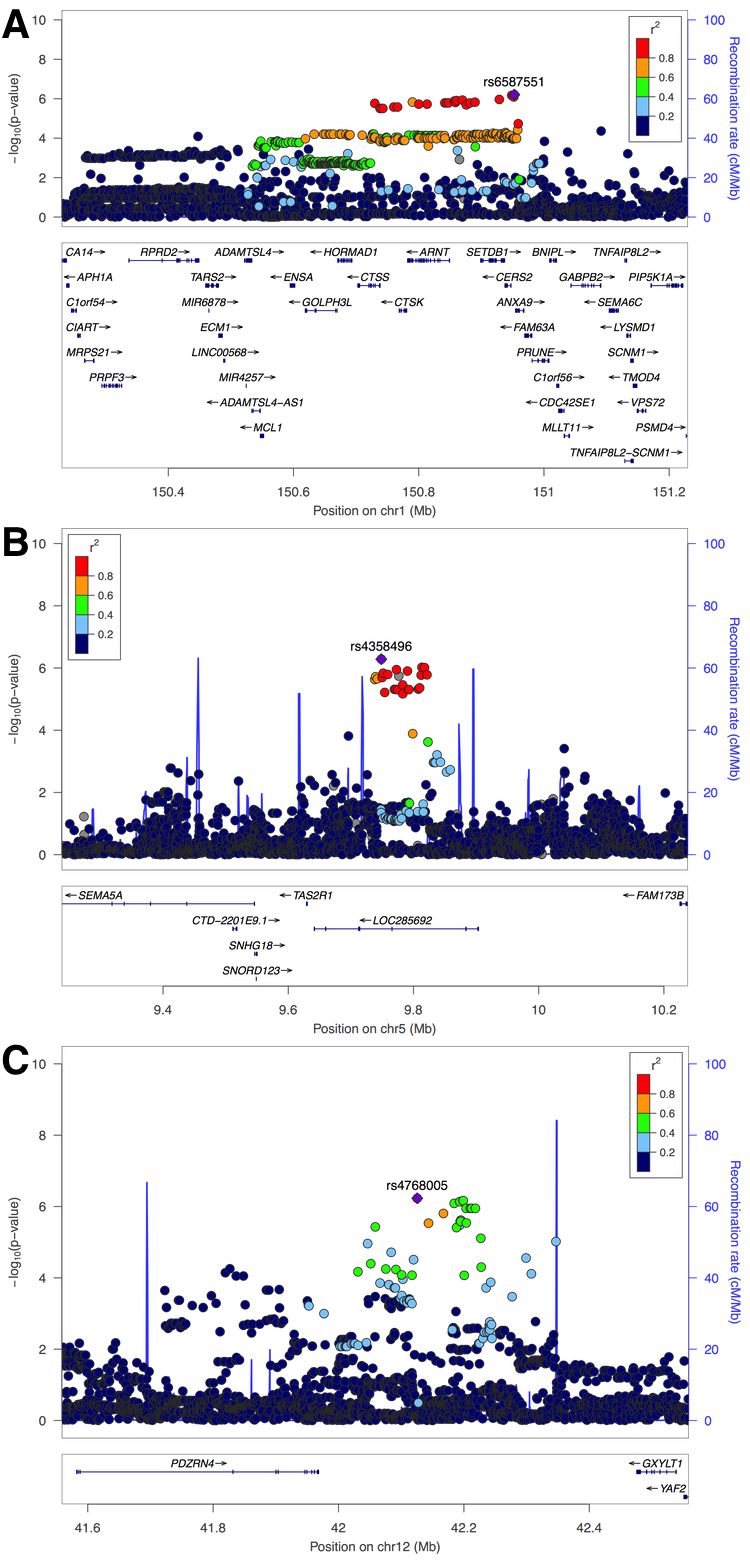

Supplement: S9 Fig — (A) Associations at locus 1q21.3; (B) Associations at locus 5p15.31; (C) Associations at locus 12q12. (PNG) [file pgen.1007973.s009.png]

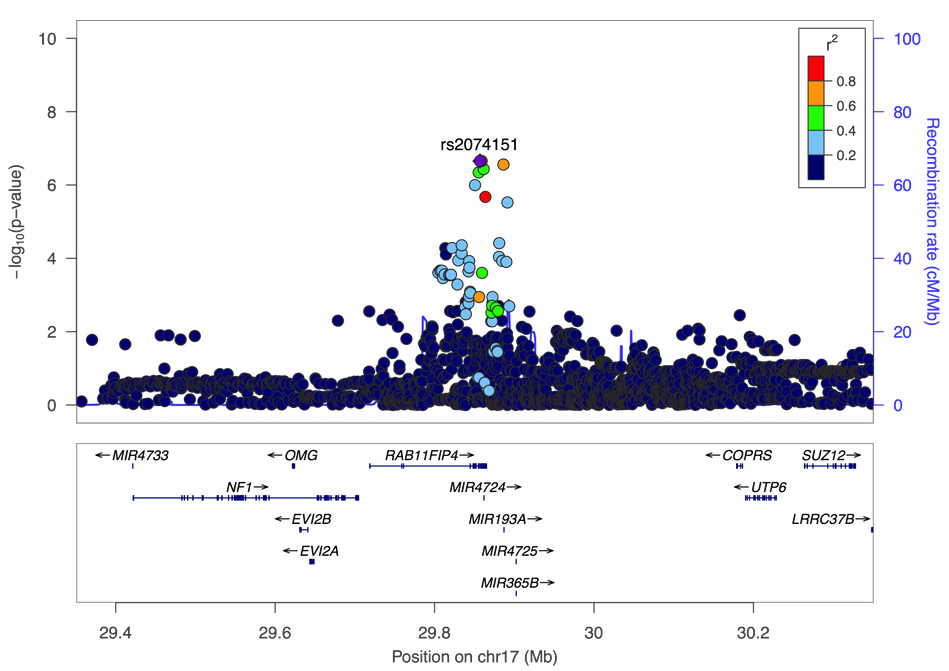

Supplement: S10 Fig — (PNG) [file pgen.1007973.s010.png]

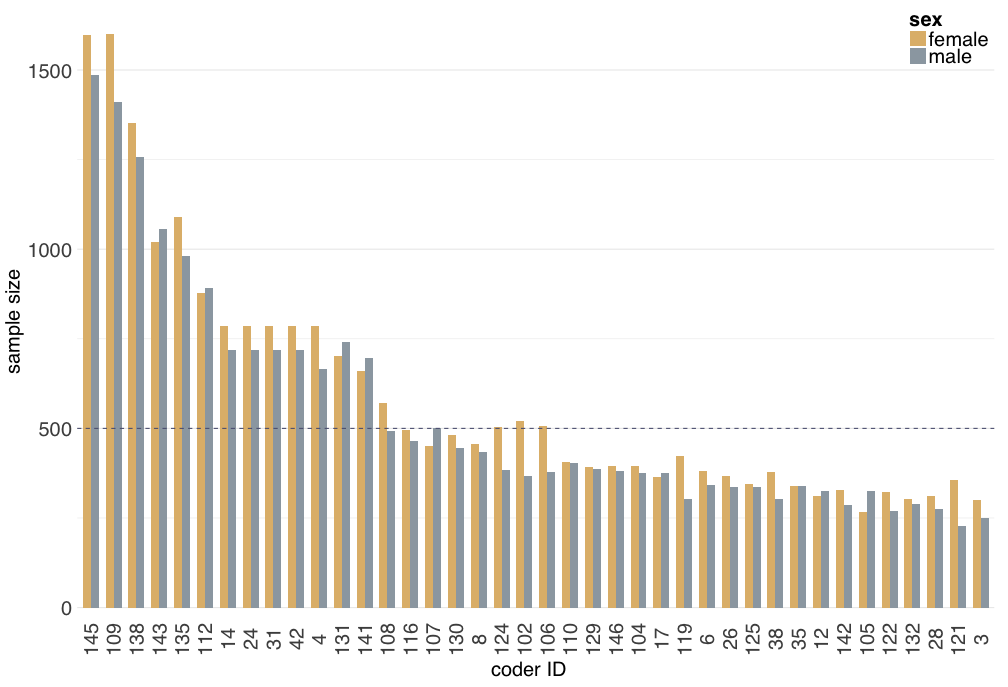

Supplement: S11 Fig — Coders who rated more than 500 male or female samples’ photos were included in association analyses based on single coders’ scoring. Coders with too few sample size were omitted from this figure. (PNG) [file pgen.1007973.s011.png]

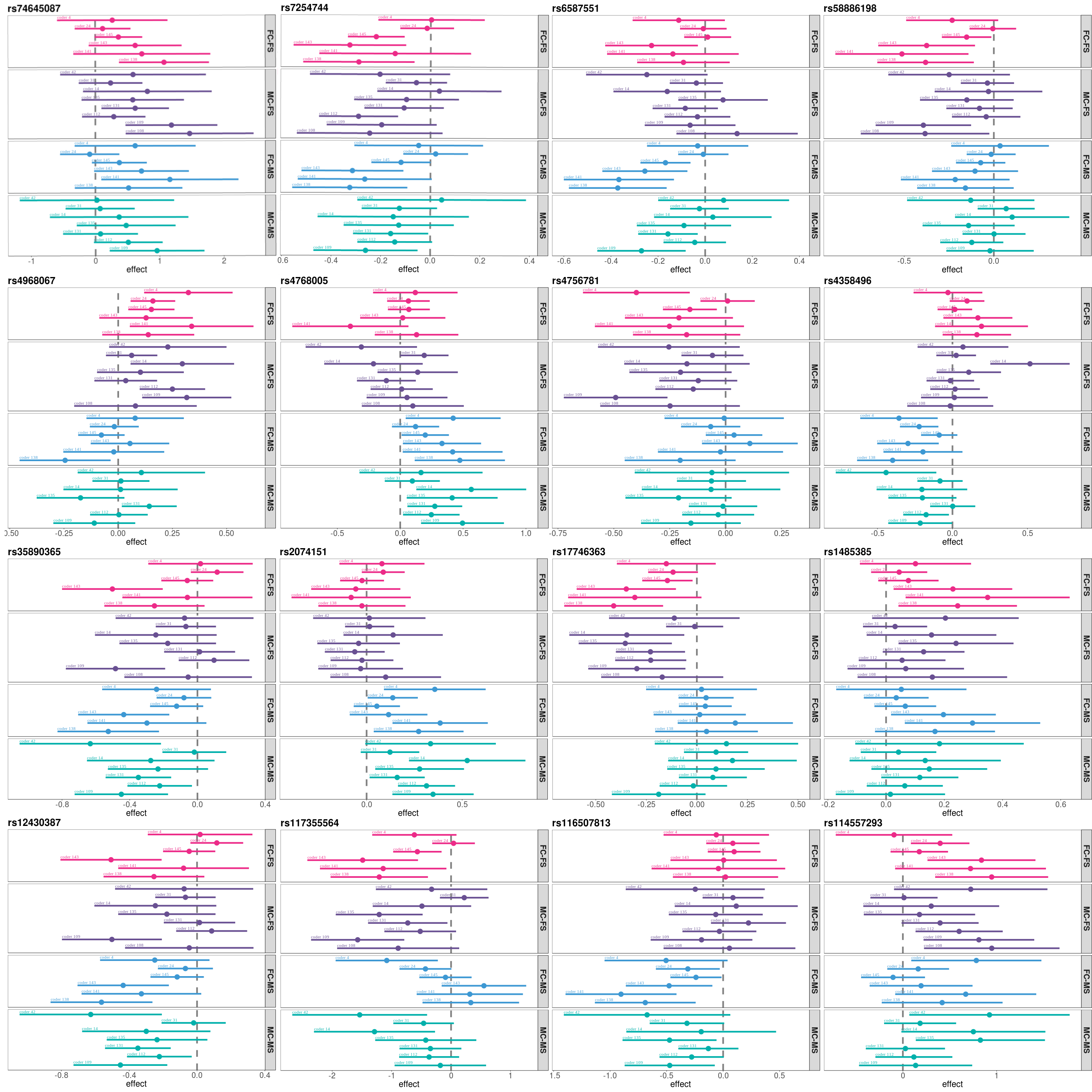

Supplement: S12 Fig — (PNG) [file pgen.1007973.s012.png]

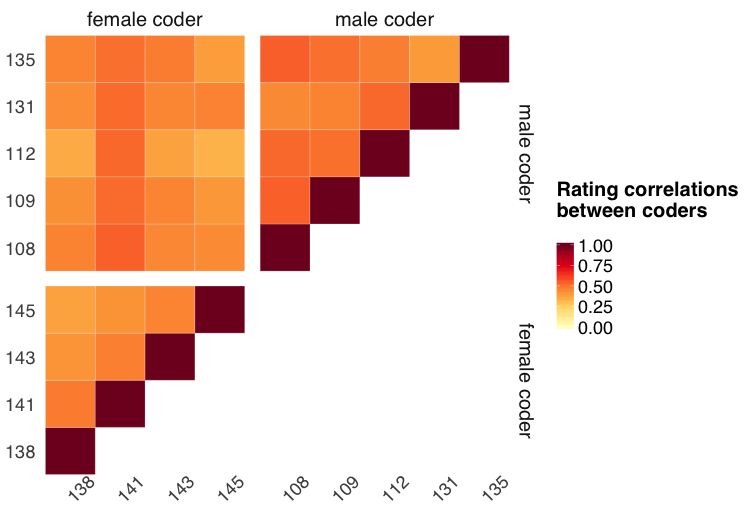

Supplement: S13 Fig — Coders who rated more than 500 photos in 2008 were analyzed. Color indicates different level of correlation. All correlations shown in the figure were statistically significant after Bonferroni correction. (PNG) [file pgen.1007973.s013.png]

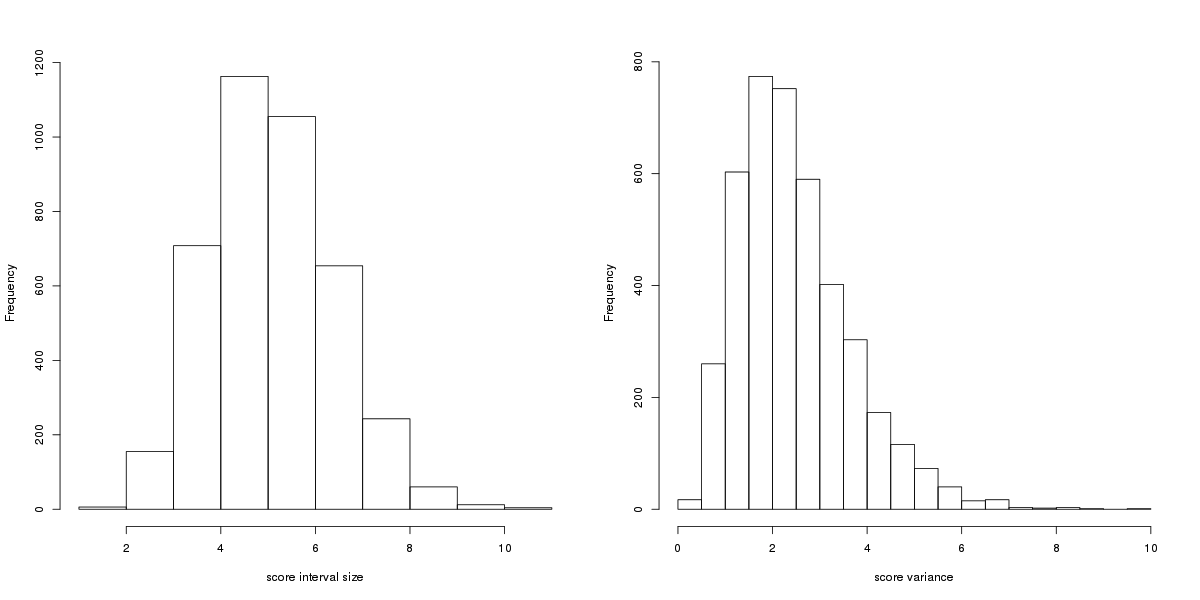

Supplement: S14 Fig — (PNG) [file pgen.1007973.s014.png]

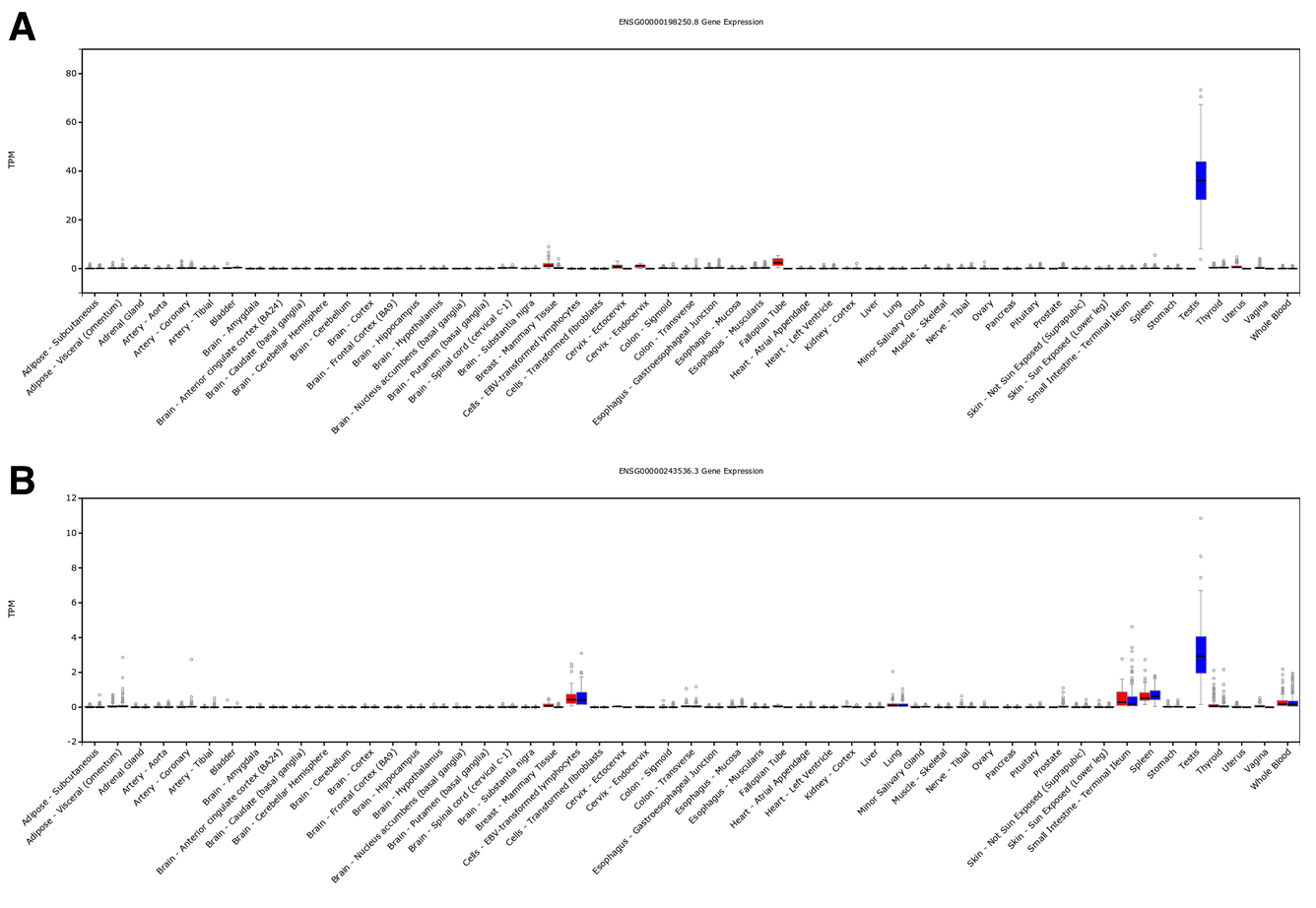

Supplement: S15 Fig — Multi-tissue gene expression profile of (A) ANTXRL and (B) ANTXRLP1 in GTEx. Blue and red boxes represent data based on male and female samples, respectively. Both ANTXRL and ANTXRLP1 have higher expression in testis than in other tissues, but the absolute expression values are low. (PNG) [file pgen.1007973.s015.png]

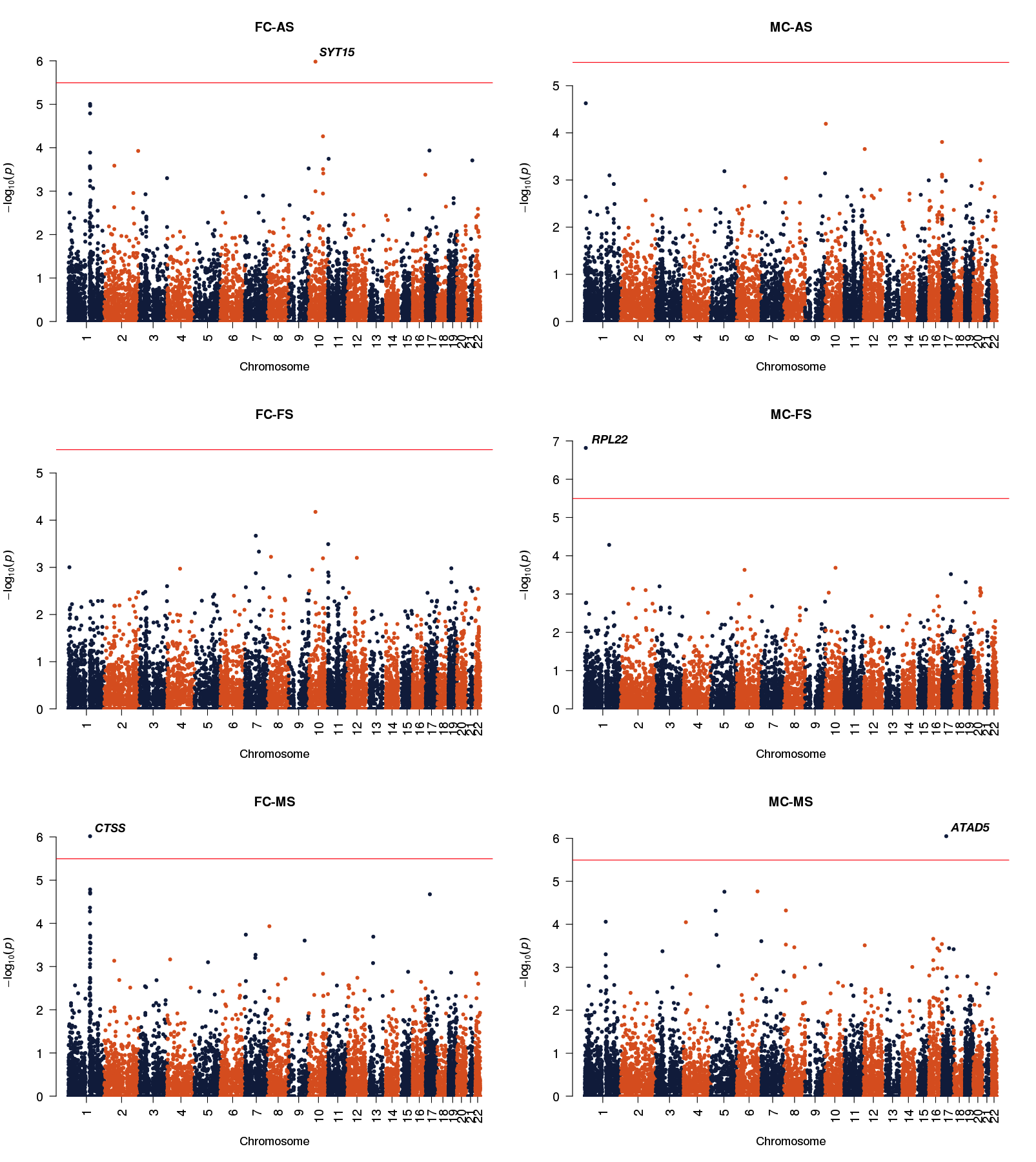

Supplement: S16 Fig — The horizontal line denotes the Bonferroni-corrected significance threshold. (PNG) [file pgen.1007973.s016.png]

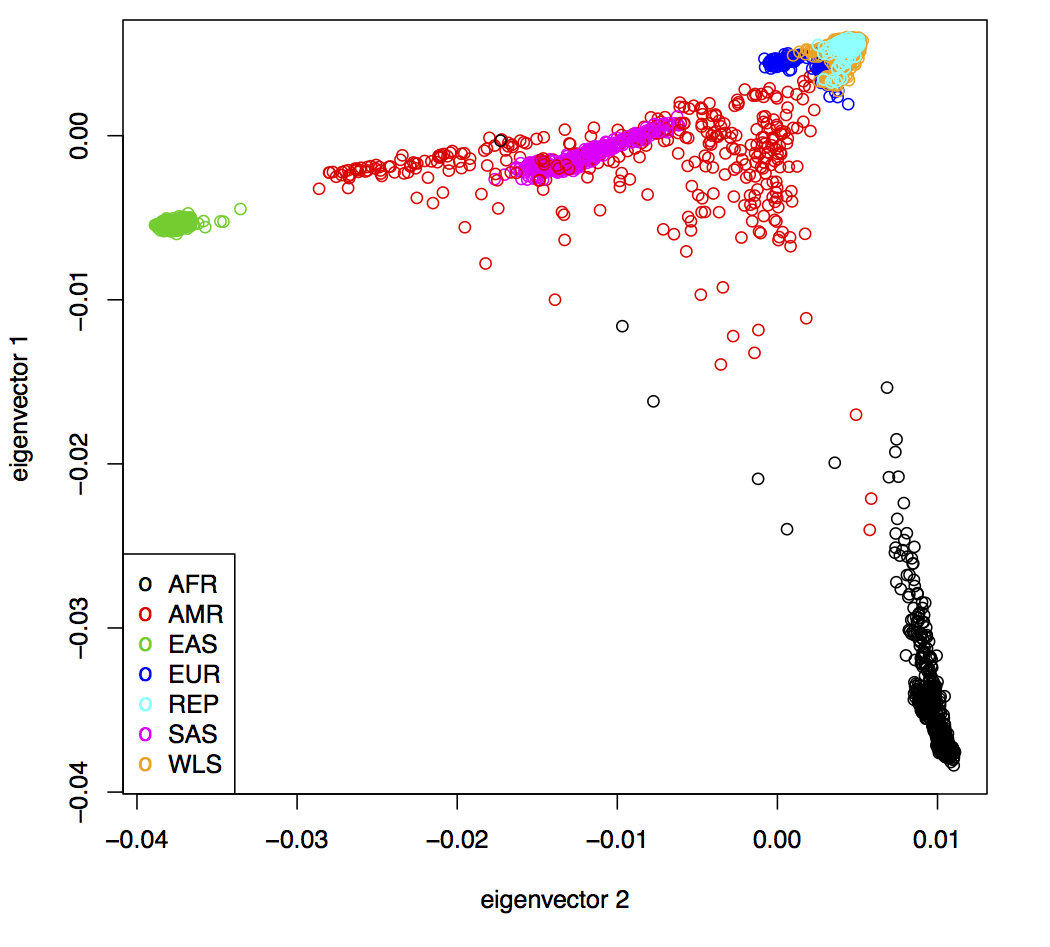

Supplement: S17 Fig — Deep blue circles represent individuals with European ancestry in 1000 Genome (EUR), orange and light blue circles represent WLS samples with self-reported European ancestry (labeled as WLS) and missing but genetically confirmed ancestry information (labeled as REP). (PNG) [file pgen.1007973.s017.png]
