# Supplementary material for: Genome-wide association study reveals sex-specific genetic architecture of facial attractiveness
Source: PLoS Genet. 2019 Apr 4;15(4):e1007973. doi: 10.1371/journal.pgen.1007973 (PMC6448826; doi:10.1371/journal.pgen.1007973)
Supplement: S11 Table — (PDF) [file pgen.1007973.s028.pdf]

S11 Table. 50 complex traits covering a variety of complex human phenotypes with publicly accessible GWAS summary statistics.

Trait	Abbreviation	N	Reference
Age at First Birth	AFB	241,781	[1]
Age at Menarche	AM	132,989	[2]
Age at Natural Menopause	ANM	69,360	[3]
Aggressive Behavior	AGG	18,988	[4]
Anorexia Nervosa	AN	14,477	[5]
Anxiety Disorder	ANX	17,310	[6]
Attention-Deficit/Hyperactivity Disorder	ADHD	53,293	[7]
Autism Spectrum Disorder	ASD	13,574	[8]
Bipolar Disorder	BIP	16,731	[9]
Birth Length	BL	28,459	[10]
Birth Weight	BW	143,677	[11]
Body Mass Index	BMI	234,069	[12]
Body Mass Index - Female	BMI-F	132,115	[12]
Body Mass Index - Male	BMI-M	104,666	[12]
Childhood BMI	CBMI	35,668	[13]
Chronotype	CHT	127,898	[14]
Cognitive Performance	COG	106,736	[15]
Depressive Symptoms	DEP	161,460	[16]
Education Years	EDU	293,723	[17]
Education Years - Female	EDU-F	182,286	[17]
Education Years - Male	EDU-M	146,631	[17]
HDL Cholesterol	HDL-C	99,900	[18]
Height	HGT	253,288	[19]
Hippocampal Volume	HV	26,814	[20]
Intracranial Volume	ICV	26,577	[21]
LDL Cholesterol	LDL-C	95,454	[18]
Major Depressive Disorder	MDD	18,759	[22]
Neuroticism	NEU	170,911	[16]
Number of Children Ever Born	NCEB	318,463	[1]
Pubertal Growth - Analysis1	PG1	13,960	[23]
Pubertal Growth - Analysis1 - Female	PG1-F	6,974	[23]
Pubertal Growth - Analysis1 - Male	PG1-M	6,986	[23]
Pubertal Growth - Analysis2	PG2	10,799	[23]
Pubertal Growth - Analysis2 - Female	PG2-F	5,756	[23]
Pubertal Growth - Analysis2 - Male	PG2-M	5,043	[23]
Pubertal Growth - Analysis3	PG3	9,228	[23]
Pubertal Growth - Analysis3 - Female	PG3-F	4,946	[23]
Pubertal Growth - Analysis3 - Male	PG3-M	4,282	[23]
Schizophrenia	SCZ	82,315	[24]
Smoking Behavior	SMK	74,053	[25]
Subjective Well-being	SWB	298,420	[16]
Tanner Stage	TS	9,916	[26]
Tanner Stage - Female	TS-F	6,147	[26]
Tanner Stage - Male	TS-M	3,769	[26]
Total Cholesterol	TC	100,184	[18]
Triglycerides	TG	96,598	[18]
Type-II Diabetes	T2D	69,033	[27]
Waist Hip Ratio	WHR	210,086	[28]
Waist Hip Ratio - Female	WHR-F	116,742	[28]
Waist Hip Ratio - Male	WHR-M	93,480	[28]

1. Barban, N., et al., *Genome-wide analysis identifies 12 loci influencing human reproductive behavior.* Nature genetics, 2016.
2. Perry, J.R., et al., *Parent-of-origin-specific allelic associations among 106 genomic loci for age at menarche.* Nature, 2014. **514**(7520): p. 92-7.
3. Day, F.R., et al., *Large-scale genomic analyses link reproductive aging to hypothalamic signaling, breast cancer susceptibility and BRCA1-mediated DNA repair.* Nat Genet, 2015. **47**(11): p. 1294-303.
4. Pappa, I., et al., *A genome-wide approach to children's aggressive behavior: the EAGLE consortium.* American Journal of Medical Genetics Part B: Neuropsychiatric Genetics, 2016. **171**(5): p. 562-572.
5. Anttila, V., et al., *Analysis of shared heritability in common disorders of the brain.* bioRxiv, 2016: p. 048991.
6. Otowa, T., et al., *Meta-analysis of genome-wide association studies of anxiety disorders.* Molecular psychiatry, 2016.
7. Demontis, D., et al., *Discovery of the first genome-wide significant risk loci for ADHD.* bioRxiv, 2017: p. 145581.
8. Lee, H., et al., *Genetic relationship between five psychiatric disorders estimated from genome-wide SNPs.* Nature Genetics, 2013. **45**(9): p. 984-94.
9. Sklar, P., et al., *Large-scale genome-wide association analysis of bipolar disorder identifies a new susceptibility locus near ODZ4.* Nature genetics, 2011. **43**(10): p. 977-U162.
10. van der Valk, R.J., et al., *A novel common variant in DCST2 is associated with length in early life and height in adulthood.* Human molecular genetics, 2014. **24**(4): p. 1155-1168.
11. Horikoshi, M., et al., *Genome-wide associations for birth weight and correlations with adult disease.* Nature, 2016. **advance online publication**.
12. Locke, A.E., et al., *Genetic studies of body mass index yield new insights for obesity biology.* Nature, 2015. **518**(7538): p. 197-206.
13. Felix, J.F., et al., *Genome-wide association analysis identifies three new susceptibility loci for childhood body mass index.* Human molecular genetics, 2015. **25**(2): p. 389-403.
14. Jones, S.E., et al., *Genome-wide association analyses in 128,266 individuals identifies new morningness and sleep duration loci.* PLoS Genet, 2016. **12**(8): p. e1006125.
15. Rietveld, C.A., et al., *Common genetic variants associated with cognitive performance identified using the proxy-phenotype method.* Proceedings of the National Academy of Sciences, 2014. **111**(38): p. 13790-13794.
16. Okbay, A., et al., *Genetic variants associated with subjective well-being, depressive symptoms, and neuroticism identified through genome-wide analyses.* Nature genetics, 2016.
17. Okbay, A., et al., *Genome-wide association study identifies 74 loci associated with educational attainment.* Nature, 2016. **533**(7604): p. 539-42.
18. Teslovich, T.M., et al., *Biological, clinical and population relevance of 95 loci for blood lipids.* Nature, 2010. **466**(7307): p. 707-713.

19. Wood, A.R., et al., *Defining the role of common variation in the genomic and biological architecture of adult human height.* Nat Genet, 2014. **46**(11): p. 1173-86.
20. Hibar, D.P., et al., *Novel genetic loci associated with hippocampal volume.* Nature communications, 2017. **8**: p. 13624.
21. Adams, H.H., et al., *Novel genetic loci underlying human intracranial volume identified through genome-wide association.* Nature neuroscience, 2016. **19**(12): p. 1569.
22. Ripke, S., et al., *A mega-analysis of genome-wide association studies for major depressive disorder.* Mol Psychiatry, 2013. **18**(4): p. 497-511.
23. Cousminer, D.L., et al., *Genome-wide association and longitudinal analyses reveal genetic loci linking pubertal height growth, pubertal timing and childhood adiposity.* Human molecular genetics, 2013. **22**(13): p. 2735-2747.
24. Ripke, S., et al., *Biological insights from 108 schizophrenia-associated genetic loci.* Nature, 2014. **511**(7510): p. 421.
25. Furberg, H., et al., *Genome-wide meta-analyses identify multiple loci associated with smoking behavior.* Nature Genetics, 2010. **42**(5): p. 441-447.
26. Cousminer, D.L., et al., *Genome-wide association study of sexual maturation in males and females highlights a role for body mass and menarche loci in male puberty.* Human molecular genetics, 2014. **23**(16): p. 4452-4464.
27. Mahajan, A., et al., *Genome-wide trans-ancestry meta-analysis provides insight into the genetic architecture of type 2 diabetes susceptibility.* Nature Genetics, 2014. **46**(3): p. 234-244.
28. Shungin, D., et al., *New genetic loci link adipose and insulin biology to body fat distribution.* Nature, 2015. **518**(7538): p. 187-96.
